# Operieren in Klinik und Praxis: Chancen, Herausforderungen und Perspektiven in der HNO-Heilkunde

**DOI:** 10.1007/s00106-025-01710-4

**Published:** 2026-01-15

**Authors:** Stefanie Jansen, Jens Peter Klussmann, Moritz Meyer, Gero Quante, Ruth Lang-Roth, Christoph Bergmann, Jan-Christoffer Lüers

**Affiliations:** 1 Klinik für HNO-Heilkunde, Kopf- und Halschirurgie, Uniklinik Köln, Medizinische Fakultät der Universität Köln, Kerpener Straße 62, 50937 Köln, Deutschland; 2 Klinik für HNO-Heilkunde, Gesichts‑, Kopf‑, Hals- und Schädelbasischirurgie, Katholisches Klinikum Koblenz, Koblenz, Deutschland; 3 Abteilung für Hals‑, Nasen‑, Ohrenheilkunde, Klinik Links vom Rhein, Köln, Deutschland; 4 RKM 740 HNO-Heilkunde Düsseldorf, Düsseldorf, Deutschland

**Keywords:** Medizinisches Fachgebiet, Chirurgische Operationsverfahren, Facharztausbildung, Ärztliche Ausbildung, Computersimulation, Medical specialty, Operative surgical procedures, Graduate medical education, Medical education, Computer simulation

## Abstract

Die operative Hals-Nasen-Ohren-Heilkunde steht in Deutschland vor einem tiefgreifenden Strukturwandel, der durch die Krankenhausreform, den Ausbau ambulanter Operationsangebote und technische Innovationen maßgeblich geprägt wird. Universitätskliniken, periphere Krankenhäuser, Praxen/Medizinische Versorgungszentren (MVZ) sowie Privatkliniken unterscheiden sich dabei deutlich hinsichtlich Ressourcen, Spezialisierung, Vergütungssystemen und Ausbildungsmöglichkeiten. Die Reform führt zu einer stärkeren Konzentration komplexer Leistungen in der HNO-Heilkunde in leistungsstarken Zentren, während standardisierbare Eingriffe zunehmend ambulant erbracht werden. Hybrid-Formen von Diagnosis Related Groups (Hybrid-DRG) und Erweiterungen des AOP-Katalogs (Ambulantes Operieren im Krankenhaus) sollen die sektorengleiche Vergütung fördern, erzeugen jedoch neue ökonomische Spannungsfelder, insbesondere für Kliniken mit hohen Vorhaltekosten. Gleichzeitig gewinnen ambulante Op.-Zentren und Praxen an Bedeutung, stehen jedoch teils vor strukturellen Nachteilen. Digitalisierung, künstliche-Intelligenz(KI)-basierte Planung, robotische Assistenz und intraoperative Bildgebung verändern das operative Arbeiten grundlegend und eröffnen neue Möglichkeiten in Präzision, Dokumentation und Ausbildung. Für die operative Weiterbildung ergeben sich neue Anforderungen: Während hochkomplexe Eingriffe zentrumsbasiert bleiben, müssen Basiseingriffe zunehmend im ambulanten Sektor erlernt werden. Dies erfordert sektorenübergreifende Rotationsmodelle, konsolidierte Curricula, digitale Simulation und eine verlässliche Förderung ambulanter Weiterbildungsstellen. Insgesamt zeigt sich, dass die Zukunft der operativen HNO-ärztlichen Versorgung in einem abgestimmten Zusammenspiel aus Klinik und Praxis, struktureller Kooperation, moderner Vergütungssystematik und technischer wie ökologischer Innovation liegt.

## Abstract (en)

Operative ear, nose and throat (ENT) medicine in Germany is facing a profound structural change, which is significantly influenced by the ongoing hospital reform, the expansion of outpatient surgery services and technical innovations. University and peripheral hospitals, practices, medical care centres and private clinics differ significantly in terms of resources, specialisation, remuneration systems and training opportunities. The reform is leading to a greater concentration of complex ENT services in high-performance centres, while standardised procedures are increasingly being performed on an outpatient basis. Hybrid forms of diagnosis-related groups (DRGs) and the *Ambulantes Operieren im Krankenhaus* (AOP; outpatient surgery) catalogue extensions are intended to promote sector-specific remuneration but are creating new economic tensions, especially for clinics with high standby costs. At the same time, outpatient surgery centres and practices are gaining in importance, but some face structural disadvantages. Digitalisation, artificial intelligence-based planning, robotic assistance and intraoperative imaging are fundamentally changing surgical work and opening up new possibilities in precision, documentation and training. This creates new requirements for surgical training: while highly complex procedures remain centre based, basic procedures must increasingly be learned in the outpatient sector. This requires cross-sectoral rotation models, consolidated curricula, digital simulation and reliable funding for outpatient training positions. Overall, the future of surgical ENT care lies in coordinated interaction between hospitals and practices, structural cooperation, modern remuneration systems, and technical and ecological innovation.

Die operative Hals-Nasen-Ohren-Heilkunde hat in den vergangenen Jahrzehnten eine bemerkenswerte Entwicklung durchlaufen. Fortschritte in der diagnostischen Bildgebung und bei minimalinvasiven Techniken sowie der Ausbau ambulanter Operationsstrukturen haben zu einer zunehmend sicheren, effizienten und patient:innenorientierten Versorgung geführt. Parallel dazu zeigen sich deutliche Unterschiede zwischen den Versorgungssektoren: Universitätskliniken, kommunale und periphere Krankenhäuser, ambulante Operationszentren und niedergelassene Facharztpraxen unterscheiden sich heute erheblich hinsichtlich ihrer infrastrukturellen Voraussetzungen, Ressourcen und Spezialisierungsgrade.

## Zentrale Zukunftsfragen

Der vorliegende Beitrag beleuchtet die aktuellen Rahmenbedingungen des operativen Arbeitens in der HNO-Heilkunde in Deutschland. Im Mittelpunkt stehen dabei die strukturellen Unterschiede zwischen Klinik und Praxis, die Auswirkungen der laufenden Krankenhausreform sowie die zunehmende Ambulantisierung chirurgischer Leistungen. Darüber hinaus werden Fragen der Versorgungsrealität und -gerechtigkeit – etwa im Hinblick auf gesetzlich und privat versicherte Patient:innen – ebenso diskutiert wie Perspektiven einer nachhaltigen und innovativen Weiterentwicklung des Fachs.

Ziel ist es, einen sachlich fundierten, differenzierten Überblick über die operative HNO-Chirurgie zu geben und zentrale Zukunftsfragen zu adressieren:

- Wie kann die operative HNO-Heilkunde unter veränderten gesundheitspolitischen und ökonomischen Rahmenbedingungen zukunftsfähig gestaltet werden?
- Wie gelingt die Integration ökologischer Nachhaltigkeit („green surgery“) und digitaler Innovationen in den klinischen und ambulanten Alltag?
- Und nicht zuletzt: Wie kann die Ausbildung des chirurgischen Nachwuchses auch in einem sich wandelnden Versorgungssystem gesichert werden?

## Operative HNO-Versorgung in einer Universitätsklinik

Operieren in einer universitären HNO-Klinik in Deutschland bedeutet im Jahr 2025, sich mit einer Vielzahl an strukturellen, organisatorischen und medizinischen Herausforderungen auseinanderzusetzen. Universitätskliniken erfüllen den Dreifachauftrag aus Patient:innenversorgung, Forschung und Lehre. Gerade im operativen Bereich der Hals-Nasen-Ohren-Heilkunde wird spürbar, wie stark aktuelle gesundheitspolitische und regulatorische Entwicklungen den klinischen Alltag prägen und wie schwierig es ist, sich zwischen Hochleistungsmedizin, Ambulantisierung und den Änderungen durch die Krankenhausreform richtig zu positionieren.

Gehen wir die einzelnen Herausforderungen durch: Die Krankenhausreform zielt auf eine stärkere Strukturierung der Krankenhauslandschaft ab, indem Versorgungsstufen und Leistungsgruppen eingeführt werden. Für Universitätskliniken bedeutet dies, dass komplexe, hochspezialisierte Eingriffe künftig noch deutlicher bei diesen konzentriert werden sollen [1]. Gleichzeitig bleibt aber die Finanzierung unsicher: Die Kliniken fordern verlässliche Rahmenbedingungen, um die hohen Anforderungen an Forschung und Lehre neben der Patient:innenversorgung zu erfüllen. Mit der Einführung von Leistungsgruppen, der Vorhaltefinanzierung sowie der anstehenden Umstellung der Vergütungssystematik wird die operative Tätigkeit im HNO-Bereich zunehmend von politischen und ökonomischen Vorgaben mitbestimmt [2].

Parallel dazu verändert die Ambulantisierung die operative Realität. Es ist anzunehmen, dass auch im HNO-Bereich vermehrt Hybrid-Formen von Diagnosis Related Groups (Hybrid-DRG) eingeführt werden, deren Vergütung dann nicht mehr von der Art der Leistungserbringung – stationär oder ambulant – abhängt. Gerade in der HNO-Chirurgie, in der viele Eingriffe auch in relativ kurzen Narkosen möglich sind, verschiebt sich ein beträchtlicher Teil der Operationen in den ambulanten Sektor [3, 4]. Patient:innen profitieren von kürzeren Aufenthalten, geringeren Infektionsrisiken und einem schnelleren Alltagseinstieg. Der Anteil ambulanter Eingriffe in der HNO-Heilkunde lag im Jahr 2010 noch bei lediglich 3,1 % aller Operationen und steigt seitdem deutlich an. Der AOP-Katalog (Katalog für Ambulantes Operieren im Krankenhaus) wurde 2025 erneut erweitert. Diese Entwicklung erfordert neue Organisationsformen, klare SOP (Standard Operating Procedures) und abgestimmte prä- und postoperative Pfade. Allerdings zeigt sich in der Ambulantisierung der Operationen ein Problem: die Finanzierung. Während stationäre Eingriffe über Fallpauschalen (DRG-System) abgerechnet werden, ist die Vergütung ambulanter Operationen oft deutlich geringer – obwohl der technische Aufwand und die Qualitätsansprüche gleichbleiben [4]. Auch Ausbildungsaspekte spielen in diesem Zusammenhang eine relevante Rolle: Eine Untersuchung von ambulanten Adenotomien in 2 Ausbildungskliniken ergab, dass Operationen von Assistenzärzten bis zu 2,4-fach länger dauerten als von Fachärzt:innen. Hierdurch ist die kostendeckende Finanzierung und damit auch die Ausbildung bedroht, zumal Abrechnungssysteme diesen Umstand nicht berücksichtigen. Die operative Weiterbildung von Assistenzärzt:innen darf nicht zur Verhandlungsmasse gesundheitspolitischer Reformen werden. Denn die heute heranwachsende Generation von HNO-Chirurg:innen wird jene sein, die die operativen Herausforderungen einer zunehmend ambulant organisierten Klinikversorgung meistern muss.

Eine weitere Herausforderung ist der anhaltende Personalmangel, im HNO-chirurgischen wie auch im Pflege- und Anästhesiebereich. Mindestbesetzungen und Personaluntergrenzen (gesetzliche Grundlage definiert und festgesetzt im Sozialgesetzbuch V und verordnet durch das Bundesministerium für Gesundheit [BMG]) sind verbindlich, was häufig zu Engpässen bei der Op.-Planung führt. Kliniken müssen deshalb ihr Op.-Management professionalisieren, Engpassalgorithmen einführen und Ausfallzeiten gezielt abfangen. Das operative Spektrum reicht in der HNO-Heilkunde von Routineeingriffen wie Adenotomien oder Septumkorrekturen bis hin zu hochkomplexen Tumoroperationen im Kopf-Hals-Bereich, mikrochirurgischen Eingriffen am Ohr und der Schädelbasis, für die gut ausgebildetes Personal essenziell ist. Eine Universitätsklinik muss eine hochspezialisierte Versorgung bereitstellen, komplizierte Fälle aus dem gesamten Versorgungsgebiet übernehmen und gleichzeitig Personal für Lehre und Forschung einsetzen [2].

Im Gegensatz zu privaten Op.-Zentren, die sich auf rentable Eingriffe konzentrieren können, muss eine Universitätsklinik das gesamte Spektrum abdecken und die technische Ausstattung auch für seltene, aufwendige und ggf. defizitäre Operationen vorhalten. Die notwendige technische Ausstattung erfordert enorme Investitionen: Ein modernes Op.-Mikroskop kostet rund 250.000–400.000 €, ein Navigationssystem für endoskopische Chirurgie 150.000–200.000 €, ein Operationslaser 80.000–120.000 € und ein Hochfrequenzbohrsystem für die Otologie 50.000–70.000 €. Diese Fixkosten sind in einer Universitätsklinik, i. d. R. in mehrfacher Ausführung, unvermeidbar, während kleinere ambulante Einrichtungen mit geringerer Ausstattung arbeiten können. Die Krankenhausreform verspricht hier Abhilfe, indem sie eine sog. Vorhaltefinanzierung vorsieht [1]. Das bedeutet, dass Kliniken für die Bereitstellung bestimmter Strukturen – etwa Notfallversorgung, Intensivmedizin oder spezialisierte Abteilungen – eine Grundvergütung erhalten, unabhängig von der Zahl der tatsächlich erbrachten Fälle. Für die HNO-Klinik einer Universitätsklinik könnte dies eine wichtige Entlastung sein.

Zusätzlich stellt sich die Frage der Ausbildung. Für junge Ärzte/Ärztinnen sind Routineeingriffe wie Tonsillektomien oder Septumoperationen unverzichtbar, um operative Fertigkeiten zu erlernen. Wenn diese Eingriffe zunehmend ambulant und außerhalb der Universitätsklinik durchgeführt werden, entsteht eine Ausbildungslücke. Damit gerät die Kernaufgabe einer Uniklinik – neben Forschung und Patientenversorgung auch die ärztliche Weiterbildung – unter Druck.

Zusammenfassend stehen die HNO-Abteilungen von deutschen Universitätskliniken vor der Herausforderung, operative Spitzenmedizin unter komplexen Rahmenbedingungen zu gewährleisten. Sie müssen einerseits die politischen Vorgaben der Krankenhausreform, die Ambulantisierung durch Hybrid-DRG und die Digitalisierung umsetzen, andererseits den Fachkräftemangel bewältigen, Patient:innensicherheit garantieren und die Weiterbildung sichern. Die geplante Krankenhausreform verschärft diese Lage, da sie auf Konzentration, Spezialisierung und Transparenz setzt, gleichzeitig aber viele Detailfragen zur Finanzierung noch offenlässt.

## Operative HNO-Versorgung in peripherer Klinik mit HNO-Hauptabteilung

Die operative HNO-Versorgung in einer peripheren Klinik mit HNO-Hauptabteilung unterscheidet sich in mehrfacher Hinsicht von den Rahmenbedingungen universitärer Zentren. Während Universitätskliniken den Dreifachauftrag aus Versorgung, Forschung und Lehre erfüllen, liegt der Fokus peripherer Häuser primär auf der klinischen Patientenversorgung und der sicherheitsorientierten Durchführung häufig vorkommender Operationen. Forschung und akademische Ausbildung treten hier – sofern kein Lehrkrankenhausstatus besteht – in den Hintergrund. Selbstverständlich gibt es bei den peripheren Kliniken große Unterschiede, und es ist sehr schwierig, diese zu einer Gruppe zusammenzufassen.

### Strukturelle und organisatorische Voraussetzungen

Periphere Kliniken verfügen i. d. R. über eine HNO-Hauptabteilung, die organisatorisch eigenständig geführt wird, jedoch eng mit den anästhesiologischen, pflegerischen und stationären Strukturen des Hauses verbunden ist. Im Unterschied zu universitären Einrichtungen ist das operative Spektrum häufig stärker auf elektive und standardisierte Eingriffe ausgerichtet – beispielsweise Tonsillektomien, Septum- und Nasennebenhöhlenoperationen, Tympanoplastiken oder die Versorgung typischer Notfälle wie Abszesse oder Nasengerüstfrakturen. Hochkomplexe oder interdisziplinäre Eingriffe, etwa an der Schädelbasis oder bei malignen Tumoren, werden meist an übergeordnete Zentren überwiesen; hier gibt es natürlich auch Ausnahmen.

### Ambulantisierung und ökonomische Herausforderungen

Die zunehmende Ambulantisierung operativer Leistungen wirkt sich auf periphere HNO-Kliniken unmittelbar aus. Viele dieser Häuser verfügen über eine gewachsene Infrastruktur für kurzstationäre oder tageschirurgische Eingriffe und sind damit prädestiniert, einen wesentlichen Teil des künftigen Hybrid-DRG-Spektrums zu übernehmen. Gleichzeitig stellt die geringere Vergütung ambulanter Leistungen bei unverändertem Personal- und Sachkostenaufwand eine erhebliche Belastung dar.

Während Universitätskliniken häufig auf Quersubventionierung und Forschungsförderung zurückgreifen können, müssen periphere Häuser den wirtschaftlichen Betrieb meist allein aus den Erlösen der Patientenversorgung sicherstellen. Damit geraten insbesondere kleinere Abteilungen in einen Zielkonflikt zwischen Effizienz und Ausbildungsauftrag.

### Personal, Ausbildung und Qualitätssicherung

Der anhaltende Fachkräftemangel betrifft periphere Kliniken in besonderem Maße. Op.-Pflege, Anästhesie und ärztlicher Dienst arbeiten häufig an Kapazitätsgrenzen. Gleichzeitig bleibt die operative Ausbildung ein zentrales Anliegen: In Lehrkrankenhäusern bestehen feste Rotationspläne, während in nichtlehrbefugten Häusern die Weiterbildung stärker praxis- als strukturorientiert erfolgt.

Der Ausbildungswert peripherer Kliniken liegt v. a. in der hohen Eigenverantwortung und der frühzeitigen operativen Tätigkeit junger Ärzt:innen. Gleichzeitig fehlen häufig die großen Fallzahlen komplexer Operationen und die interdisziplinären Fallkonferenzen, wie sie universitäre Zentren anbieten können. Die enge Kooperation mit größeren Zentren, etwa durch Rotationsprogramme, Telekonsile oder gemeinsame Fallbesprechungen, wird künftig an Bedeutung gewinnen.

## Unterschiede zwischen Unikliniken und peripheren Kliniken unter Berücksichtigung der Krankenhausreform

Seit Inkrafttreten des Krankenhausversorgungsverbesserungsgesetzes (KVVG) zum 01.01.2025 zielt die Krankenhausreform auf eine qualitätsorientierte Versorgung, die durch Vorhaltepauschalen bedingungslos stabilisiert werden soll – insbesondere abhängig von der Erfüllung definierter Leistungsgruppen auf Landesebene. Für den Bereich HNO-Heilkunde bedeutet dies: Nur Krankenhäuser, die von den Ländern eine Leistungsgruppe „HNO“ gemäß entsprechenden Qualitätskriterien zugewiesen bekommen, erhalten diese gesicherte Vorhaltefinanzierung [5].

Universitäre HNO-Kliniken profitieren deutlich von dieser Struktur: Sie verfügen über eine umfangreiche Ausstattung, spezialisierte Zentren (z. B. Tumor- oder Cochleaimplantat[CI]-Zentren), Forschung und Lehre – und erfüllen damit nicht nur die formalen Mindestanforderungen, sondern sind fast per se leistungsstarke Anbieter, die in den Landeskrankenhausplänen als versorgungsrelevant eingestuft werden [6]. Die Reform begünstigt solche Einrichtungen, indem sie – über die Vorhaltepauschalen hinaus – gezielt in Transformationsförderung investieren: Der Transformationsfonds stellt ab 2026 über eine Laufzeit von 10 Jahren bis zu 50 Mrd. € bereit, um Modernisierung und Strukturwandel zu unterstützen.

Im Gegensatz dazu stehen periphere Kliniken, die keine universitäre Anbindung haben und oft weniger spezialisierte HNO-Abteilungen besitzen. Diese Kliniken laufen Gefahr, keine Zuweisung der entsprechenden Leistungsgruppe zu erhalten – wenn sie etwa nicht genügend Fallzahlen, fachliche Struktur oder technische Ausstattung vorweisen. In Nordrhein-Westfalen gab es beispielsweise mehr Leistungsgruppenanträge als Kapazitäten. Daher wurden nur Kliniken mit nachgewiesener Leistungsstärke berücksichtigt, andere blieben außen vor [6]. Diese Nichtzuweisung kann zur Folge haben, dass die Klinik ohne Vorhaltepauschalen auskommen muss, was mittelfristig die wirtschaftliche Basis untergräbt und die stationäre HNO-Versorgung gefährden könnte.

Wie sich diese Differenzierung in der Praxis niederschlägt, zeigen die aktuellen Landesbeispiele: In Nordrhein-Westfalen wurden mit Bescheiden vom 16. Dezember 2024 landesweit 73 Versorger mit der LG (Leistungsgruppe) 24.1 „HNO“ betraut; die tatsächlichen Fallzahlen in dieser Gruppe stiegen von 94.307 (2020) auf 102.460 (2023). Zugleich betont das Land, dass nicht zugewiesene (insbesondere belegärztliche) HNO-Abteilungen nur etwa 3 % des gesamten HNO-Fallgeschehens ausmachten – ein Hinweis auf die gewollte Bündelung in leistungsstarken Standorten. Für die LG 24.1 meldeten die Bezirksregierungen in Teilräumen eine Überzeichnung der Anträge, sodass Zuweisungen gekürzt wurden; für die LG 24.2 „Cochleaimplantate“ wird die besonders restriktive Vergabe mit Überzeichnung und fallzahlbezogenen, teils befristeten Zuweisungen ausdrücklich dokumentiert. Das stärkt tendenziell universitäre CI-Zentren und schwächt periphere Anbieter ohne universitäre Einbindung [7].

Auch andere Länder ziehen nach und verschärfen die Anforderungstrias „Struktur – Qualität – Mindestumfang“: Bayern legte im Bayerischen Krankenhausplan 2025 fest, dass ab 1. Januar 2027 eine Vergütung ausschließlich für zugewiesene Leistungsgruppen vorgesehen ist. Für den Bereich HNO-Heilkunde bedeutet das: Kliniken ohne belastbare HNO-Struktur (Personal, Technik, verwandte LG) verlieren die stabile Vergütungsbasis – wiederum ein Vorteil für Universitätskliniken mit breitem Spektrum (z. B. Tumor‑, CI-, interdisziplinäre Zentren) und ein Druckpunkt für periphere Häuser, die die HNO-Heilkunde stationär nur begrenzt vorhalten können.

Somit verstärkt die Krankenhausreform die bestehende Differenzierung im HNO-Versorgungssektor. Universitätskliniken festigen ihre Rolle als regionale und überregionale HNO-Maximalversorger. Periphere Kliniken müssen sich zwischen Kooperation, Spezialisierung oder (teilweiser) Aufgabe stationärer HNO-Leistungen entscheiden – besonders, wenn sie keine Leistungsgruppe erhalten. Obwohl die Reform prinzipiell flächendeckende Versorgung sichern will, führt sie bei HNO-Leistungen aktuell zu einer Konzentration auf leistungsstarke Zentren – mit möglichen Versorgungslücken in ländlichen oder strukturschwächeren Regionen [6]. Ein aktueller „Brandbrief“ des HNO-Berufsverbands zeigt, dass die gegenwärtigen Reformpläne die HNO-Kinderchirurgie akut gefährden: Allein in Nordrhein-Westfalen wurden bereits 31 Belegabteilungen geschlossen, was mehr als 4000 ambulante Operationen betrifft [8].

## Operative Möglichkeiten in Praxen/MVZ oder ambulanten Op.-Zentren

In den vorangegangenen Abschnitten wurde bereits viel über die Ambulantisierung geschrieben, die Krankenhausreform sowie die Auswirkungen auf die Unikliniken sowie die peripheren Häuser. Aber was ist mit den ärztlichen Kolleg:innen in den Arztpraxen? Belegabteilungen werden zunehmend geschlossen, wie man es in NRW im vergangenen Jahr erlebt hat. Doch die niedergelassenen Kolleg:innen werden gebraucht, um die Vielzahl an Patient:innen – auch operativ – zu versorgen. Löhler et al. publizierten 2022, dass moderne technische Ausstattung auch in MVZ und Praxen sowie eine standardisierte Nachsorge und sichere Narkosemethoden dazu geführt haben, dass zahlreiche Eingriffe auch in niedergelassenen HNO-Praxen möglich sind [2]. Für niedergelassene Ärzte/Ärztinnen eröffnet dies nicht nur medizinisch, sondern auch ökonomisch neue Möglichkeiten, da Patient:innen wohnortnah versorgt werden können und gleichzeitig die Praxis ihr Leistungsspektrum erweitert. Auch für die Ausbildung von Weiterbildungsassistenten in der Praxis ist es wichtig, dass auch hier die chirurgische Ausbildung angeboten werden kann.

Damit ambulante Operationen in der Praxis durchgeführt werden können, sind allerdings bestimmte Voraussetzungen nötig. Neben einer adäquaten räumlichen Ausstattung mit einem Eingriffsraum oder Op.-Saal müssen strenge Hygienestandards eingehalten werden, wie sie etwa durch die Richtlinien des Robert Koch-Instituts vorgeschrieben sind. Ebenso wichtig ist die enge Kooperation mit einem Facharzt für Anästhesie, um Narkosen oder Sedierungen sicher durchführen zu können. Ein Aufwachraum mit geschultem Fachpersonal rundet die Infrastruktur ab [4]. Diese Investitionen sind nicht unerheblich, doch sie schaffen die Grundlage für ein erweitertes Leistungsangebot, das sowohl medizinisch sinnvoll als auch wirtschaftlich tragfähig sein kann.

Im ambulanten HNO-Alltag dominieren v. a. kleinere bis mittelgroße Eingriffe. Am Ohr zählen dazu etwa die Einlage von Paukenröhrchen, die besonders bei Kindern mit chronischem Paukenerguss eine Routine darstellt, sowie Korrekturen der Ohrmuschel oder die Entfernung kleiner Hautveränderungen [5]. An der Nase stehen Verfahren wie die Verkleinerung der Nasenmuscheln mittels Radiofrequenz oder Laser, kleinere Korrekturen der Nasenscheidewand und die Entfernung von Polypen im Vordergrund. Auch im Hals- und Rachenbereich werden viele Eingriffe durchgeführt, darunter die Adenotomie, also die Entfernung der Rachenmandel, oder kleinere Biopsien und Exzisionen. Hinzu kommen minimalinvasive Verfahren gegen Schnarchen, die zunehmend gefragt sind.

Ein wesentliches Handicap für ambulante Op.-Zentren, die nicht an ein Lehrkrankenhaus oder Universitätsklinikum angebunden sind, besteht in der fehlenden Möglichkeit, Ausbildungseingriffe regulär in den Op.-Ablauf zu integrieren. Jede Op.-Zeit-Verlängerung wirkt sich dort unmittelbar auf Taktung, Kostenstruktur und Fallzahlen aus. Da Ausbildungstätigkeit nicht refinanziert wird und die personelle Ausstattung kleiner ist, könnte dies einen strukturellen Wettbewerbsnachteil darstellen. Zudem ist zu berücksichtigen, dass stationäre Krankenhäuser ebenfalls ambulant operieren können. Stationäre Versorger richten bereits angegliederte ambulante Op.-Zentren ein, um ihre operative Expertise ambulant nutzbar zu machen und zusätzliche Erlöspotenziale zu erschließen. Mittelfristig ist daher von einer deutlichen Zunahme kliniknaher Op.-Zentren auszugehen, was den Wettbewerb für freistehende ambulante Einrichtungen weiter verschärfen wird.

Ein entscheidender praktischer Punkt ist die Unterscheidung zwischen gesetzlich versicherten Patient:innen und Privatpatienten. Bei gesetzlich Versicherten erfolgt die Abrechnung über den Einheitlichen Bewertungsmaßstab (EBM) [9]. Viele Standardoperationen wie Adenotomien oder Paukenröhrchen sind darin enthalten, die Vergütung ist jedoch pauschaliert und oft nicht kostendeckend, wenn man den Personalaufwand, die Geräte- und Materialkosten berücksichtigt. Moderne Techniken wie Laser- oder Radiofrequenzverfahren sind häufig nicht Bestandteil des Katalogs der Gesetzlichen Krankenversicherung (GKV) und können daher nicht regulär abgerechnet werden. Für Praxen bedeutet dies, dass sie bei gesetzlich Versicherten in einem klaren, finanziell engen Rahmen arbeiten, der durch Kooperationen mit Kliniken oder durch Selektivverträge etwas erweitert werden kann [10].

Ganz anders stellt sich die Situation bei Privatpatienten dar. Hier erfolgt die Abrechnung nach der Gebührenordnung für Ärzte (GOÄ), die Einzelleistungen detailliert erfasst. Dadurch lassen sich auch aufwendige oder innovative Verfahren abrechnen, und es besteht die Möglichkeit, je nach Aufwand den 2,3- oder 3,5-fachen Satz zu berechnen [4]. Dies eröffnet der Praxis nicht nur medizinisch größere Freiheiten, sondern macht Eingriffe ökonomisch attraktiver. Privatpatienten können eher von neuen technischen Verfahren profitieren, die GKV-Patient:innen i. d. R. nicht zur Verfügung stehen, sofern sie nicht als Selbstzahlerleistung in Anspruch genommen werden [10]. Für den Praxisinhaber sind Privatpatienten daher ein wesentlicher Faktor, um Investitionen in moderne Technik zu refinanzieren und eine nachhaltige Wirtschaftlichkeit zu sichern.

Die ökonomische Bewertung ambulanter Operationen in der HNO-Praxis zeigt somit ein gemischtes Bild. Auf der einen Seite sind GKV-Operationen wichtig, da sie i. d. R. eine kontinuierliche Auslastung von Personal und Infrastruktur gewährleisten. Sie binden Patient:innen an die Praxis und sind medizinisch unverzichtbar, auch wenn sie sich rein wirtschaftlich oft nur knapp kalkulieren lassen oder sogar teils defizitär sind [2]. Auf der anderen Seite ermöglichen Privatpatienten eine kostendeckende und teils auskömmliche Vergütung, die nicht nur den tatsächlichen Aufwand widerspiegelt, sondern auch Investitionen in moderne Verfahren ermöglicht. In der Praxis ergibt sich daraus eine Mischkalkulation: Das GKV-Segment sorgt für Kontinuität, während die Privatpatienten für die wirtschaftliche Stabilität und den Gewinn entscheidend sind.

Chancen liegen für den niedergelassenen Arzt einer Praxis darin, das angebotene Leistungsspektrum bewusst auszubauen und strategisch zu planen. Selektivverträge mit Krankenkassen können extrabudgetäre Honorierungen sichern, die Belegarzttätigkeit an einer Klinik erweitert die Möglichkeiten und erschließt zusätzliche Vergütungspfade [11]. Gleichzeitig ist es sinnvoll, in innovative Geräte zu investieren, sofern ein relevanter Anteil an Privatpatienten vorhanden ist, der die Wirtschaftlichkeit absichert. Besonders wichtig ist eine transparente Patientenaufklärung, gerade im Umgang mit GKV-Versicherten: Hier gilt es klar zu unterscheiden, was reguläre Kassenleistung ist und was als Individuelle Gesundheitsleistung (IGeL) angeboten werden kann.

Ein Blick in die Zukunft zeigt, dass die Bedeutung ambulanter Operationen in der HNO-Praxis weiter zunehmen wird. Gesundheitspolitisch wird das Prinzip „ambulant vor stationär“ zunehmend gefördert, um Kosten im System zu reduzieren [12]. Für die Praxen bedeutet dies einerseits eine Stärkung ihres Stellenwerts, andererseits aber auch die Herausforderung, wirtschaftlich tragfähige Strukturen zu schaffen. Wer medizinische Qualität mit kluger betriebswirtschaftlicher Planung verbindet, positioniert seine Praxis nicht nur als kompetente Anlaufstelle für Patient:innen, sondern auch als nachhaltig wirtschaftlich geführte Einheit im Gesundheitssystem.

So zeigt sich, dass die operative Tätigkeit in der HNO-Praxis weit mehr ist als ein Zusatzangebot. Sie ist ein integraler Bestandteil einer modernen, zukunftsorientierten Praxisführung – medizinisch unverzichtbar, ökonomisch herausfordernd und kann mit den richtigen Strategien einen Wettbewerbsvorteil darstellen. Daher sind eine angemessene Förderung und Vergütung zwingend erforderlich.

## Die §30-Privatklinik –stationäre HNO-Operationen außerhalb des GKV-Systems

Eine Privatklinik gemäß § 30 Gewerbeordnung (GewO) ist eine behördlich konzessionierte Einrichtung, die nicht in den Krankenhausplan eines Bundeslandes aufgenommen ist und ausschließlich Privatpatient:innen, Selbstzahler:innen oder Zusatzversicherte behandeln darf. Sie unterliegt der Aufsicht der zuständigen Landesbehörde, wobei insbesondere die Anforderungen der jeweiligen Landeskrankenhausverordnung und der Hygieneverordnungen erfüllt sein müssen [12].

### Strukturelle und organisatorische Voraussetzungen

Für die Zulassung nach § 30 GewO sind geeignete Räumlichkeiten, medizinisch-technische Ausstattung, qualifiziertes Fachpersonal (einschließlich Pflege und Anästhesie) sowie ein schlüssiges Hygienekonzept erforderlich. Ärztliche Leitung, Notfallmanagement, Dokumentationspflichten und ein Qualitätsmanagement nach § 135a SGB V (Sozialgesetzbuch V) müssen gewährleistet sein.

Die Konzession wird von der örtlichen Gesundheitsbehörde oder dem zuständigen Ministerium des jeweiligen Bundeslandes erteilt. Rechtlich handelt es sich um ein gewerbliches Krankenhaus im Sinne der Gewerbeordnung, das nicht an die Regelungen des Krankenhausentgeltgesetzes (KHEntgG) gebunden ist (Landesgesundheitsgesetz NRW).

### Medizinisch-operative Möglichkeiten

Im Fachgebiet der Hals-Nasen-Ohren-Heilkunde kann eine §30-Privatklinik ein geeignetes Umfeld für bestimmte stationäre Eingriffe bieten, die über das Spektrum ambulanter Operationen hinausgehen, aber keine vollstationäre Akutversorgung erfordern.

Durch die Anbindung an eine Praxis oder ein MVZ lässt sich eine durchgehende Betreuungskette von Diagnostik über Operation bis Nachsorge gestalten. Diese persönliche Kontinuität kann die Patientenzufriedenheit fördern, ersetzt aber nicht die Strukturen größerer Kliniken mit ihren interdisziplinären Ressourcen und Notfallkapazitäten.

### Abrechnung und wirtschaftliche Aspekte

Die Vergütung erfolgt in einer §30-Privatklinik vollständig nach der Gebührenordnung für Ärzte (GOÄ) und unterliegt nicht den Pauschalvergütungen des DRG-Systems. Ärztliche Leistungen, Pflege, Unterkunft und Materialkosten werden einzeln abgerechnet. Diese Form der Vergütung kann eine betriebswirtschaftlich tragfähige Kalkulation ermöglichen – vorausgesetzt, es besteht eine ausreichende Nachfrage durch Privatpatient:innen oder Selbstzahler:innen.

Gleichzeitig sind die hohen Investitionskosten und der wirtschaftliche Druck erheblich: Eine stabile Auslastung hängt stark von der regionalen Versichertenstruktur und dem Anteil an Privatversicherten ab. In Regionen mit überwiegend gesetzlich Versicherten ist der Betrieb einer solchen Klinik meist nicht wirtschaftlich darstellbar. Kooperationen mit niedergelassenen Fachärzt:innen, Anästhesiepraxen oder Rehabilitationszentren können helfen, die Auslastung zu sichern.

### Chancen und Grenzen

Das Modell der §30-Privatklinik bietet eine rechtlich etablierte Möglichkeit, bestimmte HNO-Eingriffe außerhalb des GKV-Systems anzubieten. Es kann zur Entlastung öffentlicher Kliniken beitragen und Patient:innen mit Zusatzversicherung oder besonderen Anforderungen eine individualisierte Behandlung ermöglichen.

Allerdings bleibt der Anteil solcher Einrichtungen im Versorgungssystem bislang gering. Sie können nur dort bestehen, wo die sozioökonomischen Rahmenbedingungen – insbesondere der Anteil an Privatversicherten und Selbstzahler:innen – dies zulassen. Notfallversorgung, Intensivmedizin, interdisziplinäre Kooperationen und die Ausbildung des ärztlichen Nachwuchses liegen weiterhin in der Verantwortung der öffentlichen und universitären Krankenhäuser.

Zudem birgt das privatklinische Modell strukturelle Grenzen: Es verstärkt tendenziell die Segmentierung des Versorgungssystems und bietet keine Lösung für die flächendeckende Sicherstellung operativer HNO-Versorgung. Vielmehr kann es als ergänzende Versorgungsform verstanden werden, die für ausgewählte Patientengruppen und spezialisierte Eingriffe einen zusätzlichen Handlungsspielraum schafft – ohne die zentrale Rolle der Kliniken im GKV-System zu ersetzen.

## Abrechnung von HNO-Operationen

Die Abrechnung von HNO-Operationen in Deutschland ist komplex und erfolgt je nach Versorgungssituation nach unterschiedlichen Abrechnungssystemen. Die Abrechnung von HNO-Operationen in Deutschland unterliegt einem vielschichtigen System, das sowohl die Versorgungsstruktur als auch die Art der Krankenversicherung berücksichtigt. Während stationäre Leistungen überwiegend über das DRG-System (Diagnosis Related Groups) vergütet werden, kommen im ambulanten Bereich der Einheitliche Bewertungsmaßstab (EBM) für Patient:innen der Gesetzlichen Krankenversicherung (GKV) und die Gebührenordnung für Ärzte (GOÄ) für Patienten der Privaten Krankenversicherung (PKV)/Privatpatienten zum Einsatz. Jedes System bringt spezifische medizinische, wirtschaftliche, finanzielle und rechtliche Implikationen mit sich, die sowohl für Kliniken als auch für niedergelassene Ärzte/Ärztinnen von hoher Relevanz sind. Die Unterschiede betreffen nicht nur die medizinische Kodierung, sondern auch die finanziellen Anreize, rechtlichen Rahmenbedingungen und die wirtschaftlichen Konsequenzen [9, 13].

Wesentliche abrechnungsrelevante Faktoren für die Kosten von HNO-Operationen betreffen die Art des Eingriffs (Operationstyp), die Durchführung der Leistung (stationär oder ambulant), den Schweregrad und Komplexität des Eingriffs, aber auch Nebendiagnosen und Komorbiditäten sowie das Alter und Risikoprofil der Patient:innen können eine Rolle spielen. Überdies können individuelle Nebenabreden mit Patient:innen zum Tragen kommen.

### Stationäre Operationen: Abrechnung im DRG-System

Das DRG-System ist ein durchgängiges, leistungsorientiertes und pauschalierendes Vergütungssystem, das seit 2003 verpflichtend für stationäre Krankenhausleistungen in Deutschland ist. Voraussetzung für eine Abrechnung nach dem DRG-System ist immer eine Aufnahmevereinbarung (stationärer Behandlungsvertrag) mit dem Krankenhaus. Im DRG-System erfolgt die Vergütung pauschaliert pro Fall, beeinflusst dadurch, ob sich die tatsächliche Verweildauer innerhalb einer zeitlich definierten Spanne befindet. Grundlage ist die Kodierung von Diagnosen nach ICD-10-GM (deutsche Modifikation der Internationalen statistischen Klassifikation der Krankheiten und verwandter Gesundheitsprobleme) sowie von Operationen und Prozeduren nach dem Operationen- und Prozedurenschlüssel(OPS)-Katalog. Kliniken erhalten für jede DRG eine Fallpauschale, die regional unterschiedlich sein kann, da sie vom Landesbasisfallwert abhängt. Die gesetzliche Grundlage der stationären Abrechnung regelt das SGB V, § 17b KHG (Krankenhausfinanzierung). Die Kodier- und Abrechnungsprüfung erfolgt durch den Medizinischen Dienst (MD); die gesetzliche Grundlage hierfür findet sich insbesondere in § 275 ff. SGB V, sowie durch weitere Bestimmungen im Krankenhausfinanzierungsgesetz (KHG) und durch Vereinbarungen der Selbstverwaltungspartner. Typische Problemfelder des DRG-Systems betreffen den ökonomischen Druck zur Verweildauerreduktion, die Unterfinanzierung bei Komplikationen oder langen Aufenthalten sowie Fehlanreize (beispielsweise die Selektion von Patienten/Fällen mit günstigem Case-Mix-Index) [14].

Während die Abrechnung von stationären Leistungen bei GKV-Patient:innen allein nach dem DRG-System erfolgt, folgt die Abrechnung stationärer Leistungen bei PKV-Patient:innen einem dualen System: Einerseits wird das Krankenhaus über das DRG-System gegenüber den privaten Krankenversicherungen oder Beihilfestellen vergütet, andererseits entsteht zusätzlich eine ärztliche Privatliquidation auf Basis der GOÄ. Die rechtliche Grundlage für die zusätzliche GOÄ-Abrechnung bildet das Krankenhausentgeltgesetz (KHEntgG) in Verbindung mit der Bundesärzteordnung (BÄO) und der Gebührenordnung für Ärzte (GOÄ). Nach § 17 Abs. 3 KHEntgG können Wahlleistungen im Krankenhaus gesondert vereinbart und abgerechnet werden. Dazu gehört insbesondere die ärztliche Behandlung durch den Chefarzt oder einen anderen liquidationsberechtigten Arzt, die gesondert nach den Ziffern der GOÄ in Rechnung gestellt wird. Voraussetzung für diese Form der Abrechnung ist eine individuelle Wahlleistungsvereinbarung, die der Patient vor Behandlungsbeginn schriftlich unterzeichnet. Darin erklärt er sich ausdrücklich damit einverstanden, dass bestimmte Leistungen außerhalb der Fallpauschale vergütet werden. Ohne eine solche Vereinbarung ist eine gesonderte GOÄ-Abrechnung unzulässig [13, 14].

Somit hat die GOÄ sowohl im ambulanten als auch im stationären Bereich eine Bedeutung, während der EBM ausschließlich für die ambulante Versorgung gesetzlich Versicherter vorgesehen ist. Rechtsgrundlage hierfür ist § 87 SGB V, der die Vergütung vertragsärztlicher Leistungen regelt. Im stationären Bereich gesetzlich Versicherter findet der EBM keine Anwendung. Hier greift ausschließlich das DRG-System.

### Ambulante Operationen: GKV und EBM

Bei GKV-Patient:innen läuft der Vergütungsprozess über die Kassenärztlichen Vereinigungen (KV), die als Schnittstelle zu den gesetzlichen Krankenkassen und weiteren Kostenträgern wie Knappschaft oder Berufsgenossenschaften fungieren. Damit besteht für Vertragsärzte und -kliniken keine direkte Rechnungsbeziehung zur Gesetzlichen Krankenversicherung. Dieses „Sachleistungsprinzip“ bildet die Grundlage der Gesetzlichen Krankenversicherung in Deutschland. Dabei erhalten Versicherte medizinische Leistungen direkt beim Arzt oder im Krankenhaus, ohne selbst in Vorleistung treten zu müssen. Der Leistungserbringer rechnet unmittelbar mit der Krankenkasse über die Kassenärztliche Vereinigung ab. Für den Patienten entstehen somit keine direkten Kosten, außer gesetzlich vorgesehener Zuzahlungen.

Ambulante Operationen werden bei GKV-Patient:innen nach dem EBM, in dem alle abrechnungsfähigen Operationen mit Ziffern und Punktwerten kodiert sind, erfasst. Die Vergütung ambulanter Operationen im EBM unterscheidet sich jedoch von der Abrechnung klassischer Einzelleistungen. Im Bereich der ambulanten Chirurgie und HNO-Operationen wird nicht jede einzelne Ziffer unmittelbar mit einem festen Punktwert vergütet, sondern der Eingriff wird einer Operationskategorie zugeordnet. In diesem im Anhang 2 des EBM verankerten Kategoriesystem sind ambulante HNO-Eingriffe bei GKV-Versicherten in 7 Operationsstufen (N1 bis N7) eingeteilt, deren Schwierigkeit, Dauer, Ressourcenaufwand und Komplexität mit steigender Kategorie zunimmt. Die Höhe der Vergütung innerhalb dieser Kategorien ist bundeseinheitlich definiert, wird jedoch regional durch die Kassenärztlichen Vereinigungen anhand des jeweiligen Euro-Umrechnungsfaktors konkretisiert [9].

Im Kern orientiert sich die Abrechnung ambulanter Operationen bei GKV-Versicherten an der Kombination von OPS-Codes und EBM-Ziffern. Der OPS-Code beschreibt die medizinische Prozedur und ist damit der Ausgangspunkt für die Abbildung im EBM. So „triggert“ beispielsweise der OPS-Code 5‑285.0 (Adenotomie [ohne Tonsillektomie]: Primäreingriff) die EBM-Ziffer 31231 (bei ambulanter Operation) oder die EBM-Ziffer 36231 (belegärztliche Operation). Beide EBM-Ziffern sind im Anhang 2 des EBM der Kategorie N1 zugeordnet, wodurch festgelegt ist, in welchem Honorarkorridor der Eingriff vergütet wird (Tab. 1; [9]).

**Tab. 1:** Beispiel der systematischen Kodierung typischer ambulanter (nicht belegärztlicher) HNO-Operationen nach dem Anhang 2 EBM

Op.-Bezeichnung	OPS-Ziffer	Kategorie	EBM-Ziffern		
			Op.-Leistung	Postoperative Überwachung	Postoperative Nachsorge
Adenotomie (ohne Tonsillektomie): Primäreingriff	52-85.0	N1	31231	31502	31657
Myringoplastik (Tympanoplastik Typ I): endaural	5‑194.0	N2	31232	31503	31659
Plastische Rekonstruktion des Nasenseptums: Austauschplastik	5‑214.70	N3	31233	31504	31659
Radikale zervikale Lymphadenektomie (Neck-Dissection): radikal: 4 Regionen	5‑403.10	N4	31234	31504	31661

*EBM* Einheitlicher Bewertungsmaßstab, *OPS* Operationen- und Prozedurenschlüssel

Neben der eigentlichen Operationsziffer können im Rahmen ambulanter Eingriffe auch noch weitere Leistungen abgerechnet werden, die ihrerseits wiederum durch die Kategorie definiert sind. Dazu zählen insbesondere die postoperative Überwachung (abrechenbar für eine definierte Überwachungsdauer im Anschluss an die Op.) und Nachsorgeleistungen, die Kontrolluntersuchungen und medizinische Betreuung im postoperativen Verlauf abbildet.

In regelmäßigen Intervallen – üblicherweise am Ende eines Quartals – übermitteln die Praxen und Kliniken die genannten Daten der durch sie durchgeführten ambulanten Operationen an die zuständige KV. Dort werden die Angaben geprüft, aufbereitet und anschließend gegenüber den Krankenkassen geltend gemacht. Die KV verteilt die bei den Krankenkassen eingeforderten Gelder anschließend an die Praxen oder Kliniken, die ambulante Operationen durchführen.

### Ambulante Operationen: PKV und GOÄ

Bei PKV-Patient:innen werden ambulante Operationen nach der GOÄ vergütet. PKV-Patient:innen erhalten ihre Rechnung unmittelbar vom behandelnden Arzt oder über ein Abrechnungsinstitut und müssen diese Rechnung primär begleichen. Hierzu empfiehlt sich ein schriftlich formulierter Behandlungsvertrag. Der Patient reicht die Rechnung anschließend bei seiner privaten Krankenversicherung oder Beihilfestelle ein und stellt einen Erstattungsantrag, was als Kostenerstattungsprinzip bezeichnet wird. Für die Abrechnung spielt hierbei auch die Unterscheidung zwischen medizinisch indizierten Eingriffen und kosmetischen Operationen eine Rolle, da medizinisch notwendige Operationen gemäß § 4 Nr. 14 UStG von der Umsatzsteuer befreit sind, da sie der Heilbehandlung dienen. Kosmetische Operationen ohne medizinische Indikation hingegen gelten als umsatzsteuerpflichtige Leistungen. Hier ist der Operateur:in verpflichtet, zusätzlich zur GOÄ-Gebühr die Umsatzsteuer in Rechnung zu stellen. Erfolgen medizinisch indizierte und kosmetische Operationen im gleichen Eingriff, so empfiehlt sich eine getrennte Rechnungsstellung dieser Leistungen [15].

Besonderheiten sind auch bei Patient:innen aus dem Ausland zu beachten. In aller Regel besteht hier ein direktes Vertragsverhältnis zwischen Arzt bzw. Klinik und dem Patienten. Der Behandlungsvertrag richtet sich nach deutschem Zivilrecht, und die Vergütung erfolgt üblicherweise auf Grundlage der GOÄ.

Ein temporärer Übertritt von GKV-Versicherten in den nach GOÄ vergüteten privaten Leistungssektor ist auf unterschiedlichen Wegen möglich. So etwa nach dem Kostenerstattungsmodell per Antrag an die Krankenkasse (Wechsel vom Sachleistungs- in das Kostenerstattungsprinzip), als Selbstzahler und für einzelne individuelle Gesundheitsleistungen.

#### Kostenerstattungsmodell

Für GKV-Versicherte gibt es grundsätzlich die Möglichkeit, vom üblichen Sachleistungsprinzip (der Arzt rechnet direkt mit der Krankenkasse ab) in das sog. Kostenerstattungsprinzip zu wechseln. Rechtsgrundlage dafür ist § 13 SGB V. In diesem Modell bezahlt der Patient zunächst selbst die Behandlung (z. B. nach GOÄ) und erhält anschließend von seiner Krankenkasse einen Teil der Kosten zurück. Die Rückerstattung erfolgt dabei jedoch nur in der Höhe, wie sie bei einer Abrechnung nach dem EBM vorgesehen wäre. Ein solcher Wechsel muss schriftlich bei der Krankenkasse beantragt werden und gilt jeweils für mindestens ein Quartal. Zwar hat jeder Versicherte das Recht, dieses Prinzip zu wählen, aber die Kostenübernahme durch die Krankenkasse ist keine Pflichtleistung, sondern eine Kann-Regelung. Das heißt: Die Kasse darf entscheiden, ob und in welchem Umfang sie die Kosten erstattet [9].

#### Selbstzahler

GKV-Patient:innen haben überdies die Möglichkeit, ambulante Operationen als Selbstzahler anzunehmen. In diesem Fall verzichten GKV-Patient:innen auf eine Abrechnung über den EBM und die KV und treten stattdessen in ein direktes privatrechtliches Behandlungsverhältnis mit dem Arzt oder der Klinik ein. Der Patient erhält dann eine Privatliquidation, die er vollständig selbst zu tragen hat, da eine Erstattung durch die GKV ausgeschlossen ist. Dieses Modell kommt insbesondere dann in Betracht, wenn Patient:innen eine bestimmte Behandlungsform wünschen, die nicht zum Leistungskatalog der GKV gehört, oder wenn sie bewusst eine Behandlung durch einen Chefarzt oder Spezialisten nach GOÄ-Vergütung bevorzugen. Rechtlich ist dabei zwingend erforderlich, dass der Patient vorab eine schriftliche Selbstzahlervereinbarung unterschreibt. Diese muss klarstellen, dass die Behandlung nicht als Kassenleistung, sondern ausschließlich als private Leistung nach GOÄ erfolgt [9, 13].

#### Individuelle Gesundheitsleistungen

Neben den über den EBM abgedeckten Leistungen können bei gesetzlich Versicherten auch Individuelle Gesundheitsleistungen (IGeL) eine Rolle spielen. Darunter versteht man medizinische Maßnahmen, die aus Sicht des Patienten wünschenswert oder medizinisch sinnvoll sein können, jedoch nicht zum Leistungskatalog der gesetzlichen Krankenversicherung gehören. Typische Beispiele von Operationen mit IGeL-Charakter im HNO-Fachgebiet wären die Schlafendoskopie in Sedierung oder Vollnarkose zur differenzierten Diagnostik des Schnarchens oder des obstruktiven Schlafapnoesyndroms, sofern keine medizinische Notwendigkeit nach GKV-Kriterien vorliegt, minimalinvasive Eingriffe an den Nasenmuscheln (z. B. Radiofrequenztherapie zur Verbesserung der Nasenatmung), wenn die Indikation primär funktionell-kosmetisch und nicht medizinisch zwingend ist, oder Laseroperationen im Kehlkopfbereich bei funktionellen Stimmstörungen ohne Krankheitswert, die keine EBM-Abdeckung finden. Derartige Eingriffe müssen vorab mit dem Patienten besprochen und als IGeL-Leistungen schriftlich vereinbart werden. Abgerechnet wird i. d. R. nach GOÄ. Damit tragen Patient:innen die Kosten selbst, sofern keine medizinische Notwendigkeit im Sinne der GKV anerkannt ist [9, 13, 16].

#### PKV-System

Grundsätzlich werden analog zu konservativen ärztlichen Leistungen im PKV-System auch ambulante Operationen nicht als Pauschalbetrag vergütet. Stattdessen setzt sich auch hier die Rechnung aus vielen GOÄ-Einzelposten zusammen. So können in die spätere Rechnung einer ambulanten Operation auch ärztliche Leistungen beinhaltet sein, die rund um die Operation anfallen, so etwa Beratungsleistung und Aufklärungsgespräch sowie die klinische Vor- und Nachuntersuchung (auch noch am Op.-Tag). Erstattungsfähig sind zudem Einmal- und Verbrauchsmaterialien, soweit sie über eine „Auslagenregelung“ (§ 10 GOÄ) abgerechnet werden können. Beispiele: Kanülen, Fäden, Verbandmaterial, Implantate. Nicht abrechnungsfähig sind allgemeine Praxiskosten, also Betriebskosten wie Miete, Energie, Grundausstattung und auch die Sterilisation von Instrumenten – diese gilt als Praxisinfrastruktur und ist in den GOÄ-Leistungen einkalkuliert. Auch bildgebende Verfahren (Endoskopie, Sonographie) oder Laborleistungen, wenn im Zusammenhang mit der Op. erforderlich und nach GOÄ berechenbar. Die Anästhesieleistung hingegen erhält eine eigene GOÄ-Ziffer, die jedoch i. d. R. durch den Leistungserbringer (den Anästhesisten) selbst in eigener Rechnung an den Patienten zur Geltung gebracht wird. Für operative Eingriffe an Sonn- oder Feiertagen oder nachts gibt es zudem Zuschläge (A–K).

## Hybrid-DRG in der HNO-Versorgung

### Sektorenübergreifendes Vergütungsmodell

Mit der Einführung der sog. Hybrid-DRG zum 1. Januar 2024 gemäß § 115f SGB V hat der Gesetzgeber ein neues, sektorenübergreifendes Vergütungssystem geschaffen, das einen Paradigmenwechsel in der Krankenhaus- und Praxislandschaft markiert. Ziel der Hybrid-DRG ist es, eine sektorengleiche Vergütung für bestimmte Eingriffe zu etablieren, die sowohl ambulant als auch stationär erbracht werden können, und damit die im Gesundheitswesen häufig beklagte Trennung zwischen ambulanter und stationärer Versorgung zu überwinden (Spitzenverband stationäre Versorgung). Während bisher viele operative Leistungen im Fachgebiet der Hals-Nasen-Ohren-Heilkunde aufgrund von DRG-Rahmenbedingungen ausschließlich stationär durchgeführt wurden, soll die Hybrid-DRG die Verlagerung dieser Eingriffe in den ambulanten Bereich fördern. Damit soll der Grundsatz „ambulant vor stationär“ auch wirtschaftlich wirksam werden [9].

#### Chancen

Für die HNO-Chirurgie eröffnet dieses neue Modell prinzipiell bedeutende Chancen, da es die Vergütungsunterschiede zwischen operativen Praxen, MVZ, ambulanten Op.-Zentren und Krankenhäusern verringern kann. Eine einheitliche Fallpauschale für ausgewählte Operationen könnte Investitionen in ambulante Op.-Infrastruktur begünstigen und die Planungssicherheit erhöhen. Insbesondere kleinere bis mittelgroße Eingriffe – etwa Adenotomien, Tonsillotomien oder Septumplastiken – lassen sich unter standardisierten Bedingungen sicher ambulant durchführen und wären geeignete Kandidaten für eine sektorenübergreifende Vergütung. Damit könnten Krankenhäuser entlastet und zugleich wohnortnahe Versorgungsangebote gestärkt werden.

#### Herausforderungen

Allerdings weist die derzeitige Ausgestaltung der Hybrid-DRG erhebliche Herausforderungen und Kritikpunkte auf. In ihrer Stellungnahme zum Referentenentwurf der Hybrid-DRG-Verordnung bemängelt die Deutsche Gesellschaft für Hals-Nasen-Ohren-Heilkunde, Kopf- und Hals-Chirurgie (DGHNO-KHC), dass das Modell bislang nicht ausreichend auf die spezifischen Anforderungen der HNO-Chirurgie eingeht (Bundesministerium für Gesundheit). So differenziere die Hybrid-DRG zu wenig zwischen Einrichtungen mit hohen Vorhaltekosten – etwa Krankenhäusern mit Notfall- und Intensivstrukturen – und ambulanten Op.-Zentren, deren Fixkosten geringer, aber deren Fallzahlen ebenfalls begrenzt seien. Zudem seien viele für die HNO-Heilkunde relevante Eingriffe bislang nicht im Hybrid-DRG-Katalog enthalten, der sich derzeit primär auf andere Fachgebiete wie Allgemein‑, Viszeral- oder orthopädische Chirurgie konzentriert.

Darüber hinaus kritisieren Fachgesellschaften, dass der derzeitige Vergütungssatz in den Pilot-Hybrid-DRG die tatsächlichen Kosten ambulanter Operationen in vielen Fällen nicht kostendeckend abbildet. In HNO-Einrichtungen mit eigenem Anästhesieteam, Pflegepersonal und Nachsorgestrukturen bestehen deutliche Unterschiede zu rein ambulanten Strukturen ohne eigene Infrastruktur. Auch postoperative Komplikationen, die eine stationäre Nachbeobachtung erforderlich machen können, sind im Hybrid-DRG-System bislang nur unzureichend berücksichtigt. Hier besteht die Gefahr einer Risikoselektion: Einfache Fälle könnten ambulant verlagert werden, während Patient:innen mit komplexeren Erkrankungen weiterhin stationär verbleiben – was eine Ungleichverteilung zulasten der Krankenhäuser und eine Wettbewerbsverzerrung zur Folge hätte.

#### Ambivalentes Bild

Für HNO-Praxen und ambulante Op.-Zentren ergibt sich daraus ein ambivalentes Bild: Einerseits bietet die Hybrid-DRG eine Perspektive für eine gerechtere und planbarere Vergütung ambulanter Operationen, andererseits bleibt die konkrete betriebswirtschaftliche Umsetzung unsicher. Entscheidend wird sein, ob der Hybrid-DRG-Katalog künftig um häufige HNO-Eingriffe – wie die Adenotomie, Tonsillotomie oder Nasenmuschelverkleinerung – erweitert wird. Mehrere Fachgesellschaften und Berufsverbände, darunter der Deutsche Berufsverband der HNO-Ärzte, fordern eine rasche Integration dieser Eingriffe in den Vergütungskatalog, um die Ambulantisierung in der HNO-Heilkunde tatsächlich zu fördern [17].

Langfristig kann das Hybrid-DRG-System nur dann erfolgreich sein, wenn es sowohl medizinische Qualität als auch wirtschaftliche Realität in den verschiedenen Versorgungssektoren abbildet. Für die HNO-Chirurgie bedeutet dies eine konsequente Einbeziehung der Fachgesellschaften in die Weiterentwicklung der Bewertungsgrundlagen und eine differenzierte Berücksichtigung der spezifischen Kosten- und Strukturbedingungen ambulanter und stationärer Leistungserbringer [17–19].

## Digitalisierung und Innovation im HNO-OP

Die Hals-Nasen-Ohren-Chirurgie kann in besonderem Maße von der Digitalisierung profitieren. Komplexe anatomische Strukturen, die Notwendigkeit hochpräziser Eingriffe sowie eine enge Verzahnung mit bildgebenden Verfahren prädestinieren das Fach für den Einsatz digitaler Technologien. Der größte Nutzen der Digitalisierung zeigt sich dabei direkt im operativen Setting – also im OP selbst sowie in der prä- und intraoperativen Phase [20–23].

### Künstliche Intelligenz und digitale Op.-Planung

Hochauflösende Computertomographie(CT)- und Magnetresonanztomographie(MRT)-Daten liefern die Grundlage für die präzise Operationsplanung. Mithilfe von durch künstliche Intelligenz (KI-)gestützten Algorithmen können anatomische Strukturen automatisch segmentiert und Risikobereiche identifiziert werden [24, 25]. Solche Systeme unterstützen Chirurg:innen bei der individuellen Planung, etwa bei der optimalen Elektrodeninsertion bei Cochleaimplantationen [26–28] oder der Risikoeinschätzung bei Tumorresektionen.

In der Otologie und Schädelbasischirurgie werden zunehmend patientenspezifische 3‑D-Modelle verwendet, die eine präoperative Simulation des Eingriffs erlauben [29, 30]. Diese können über additive Fertigung (3-D-Druck) oder virtuelle Simulation in der Planungssoftware realisiert werden. Dadurch werden individuelle anatomische Variationen besser berücksichtigt, was Sicherheit und Effizienz steigert.

### Robotische Assistenzsysteme

Robotische Systeme haben in der HNO-Chirurgie in den letzten Jahren Einzug gehalten, insbesondere in Form der transoralen roboterassistierten Chirurgie (TORS). Diese Verfahren eröffnen minimalinvasive Zugänge zum Oropharynx und Larynx, die konventionell schwer erreichbar sind [31]. Auch in der Otologie kommen robotische Systeme zum Einsatz – beispielsweise zur präzisen Bohrung im Felsenbein oder zur robotergestützten Elektrodeneinführung bei Cochleaimplantaten [32]. Klinische Erfahrungen zeigen eine höhere Präzision, eine geringere Traumatisierung des Gewebes und potenziell eine Reduktion postoperativer Komplikationen. Herausforderungen bestehen weiterhin in den hohen Anschaffungskosten, dem Schulungsaufwand und der Integration in bestehende Op.-Prozesse. Dennoch gilt Robotik als einer der wichtigsten Treiber zukünftiger Op.-Entwicklung [33].

### Intraoperative Bildgebung und Navigation

Moderne intraoperative Bildgebungssysteme, etwa mobile CT- oder MRT-Geräte, ermöglichen eine direkte Kontrolle des Operationsergebnisses noch während des Eingriffs – z. B. bei Cochleaimplantationen [34] oder komplexen Tumorresektionen [35].

In Kombination mit Navigationssystemen entsteht eine Art „GPS für den OP“, das Chirurg:innen eine exakte Orientierung bietet. Durch hochauflösende, teils 3‑D-fähige Endoskope [36], Fluoreszenzbildgebung [37] oder optische Kohärenztomographie (OCT) [38] kann Tumorgewebe intraoperativ besser von gesundem Gewebe abgegrenzt werden. Solche Verfahren erhöhen die Präzision und tragen zur onkologischen Sicherheit bei.

### Digitale Visualisierung und Ergonomie

Digitale Op.-Mikroskope mit eingeblendeten Bilddaten und Vitalparametern erweitern den Informationshorizont während der Operation. Es gibt Systeme, die eine Kopfsteuerung über ein Display erlauben und so die Ergonomie und Arbeitshaltung im OP verbessern [39, 40].

Die Integration von Augmented-Reality-Technologien eröffnet die Möglichkeit, präoperative Bilddaten direkt ins Sichtfeld der Operateure einzublenden. Dadurch können anatomische Landmarken, Tumorränder oder Implantatpositionen virtuell sichtbar gemacht werden – ein entscheidender Vorteil bei komplexen Eingriffen [22].

### Digitale Dokumentation und Prozessintegration

Ein oft unterschätzter Aspekt der Digitalisierung betrifft die automatisierte Op.-Dokumentation. Digitale Systeme erfassen Instrumentennutzung, Zeitstempel, Implantate und Verbrauchsmaterialien in Echtzeit. Diese Daten können unmittelbar in die elektronische Patientenakte übertragen werden und dienen sowohl der Qualitätssicherung als auch der ökonomischen Analyse [41, 42]. Langfristig könnte die Kombination aus Dokumentation, Bilddaten und intraoperativen Sensorinformationen zu einer standardisierten, datengestützten Bewertung operativer Qualität führen [43].

### Schulung, Simulation und Ausbildung

Digitale Technologien eröffnen neue Wege in der chirurgischen Aus- und Weiterbildung. Virtuelle Trainingsmodule und immersive Simulationen ermöglichen realitätsnahe Übungsszenarien – insbesondere in anatomisch engen Bereichen wie der Nase, der Schädelbasis oder dem Mittelohr [44]. KI-basierte Feedbacksysteme können dabei helfen, Fehlerquellen zu erkennen und operative Fähigkeiten gezielt zu verbessern [45, 46].

Fazit: Die Digitalisierung verändert den HNO-OP grundlegend. Robotik, intraoperative Bildgebung, KI-gestützte Planung und Augmented Reality führen zu einer neuen Qualität von Präzision und Sicherheit. Zugleich bleiben Kosten, technische Integration und Datenschutz Herausforderungen, die einer breiten Implementierung derzeit noch Grenzen setzen.

Die Zukunft des Operierens in Klinik und Praxis liegt in der intelligenten Verbindung von Mensch und Technik – mit dem Ziel, operative Eingriffe sicherer, effizienter und patientenindividueller zu gestalten.

## Operieren in Praxis und Klinik international

Operative Leistungen werden in Deutschland traditionell überwiegend stationär erbracht, auch wenn seit Jahren der Anteil ambulanter Eingriffe steigt. Im internationalen Vergleich zeigt sich, dass Deutschland eine der höchsten Krankenhausbettenzahlen und stationären Operationsraten unter den OECD-Ländern hat [47, 48]. Ambulantes Operieren ist rechtlich möglich und wird sowohl in Kliniken als auch in freien Op.-Zentren oder Praxen durchgeführt. Dennoch liegt der Anteil ambulanter Operationen unter dem Niveau vergleichbarer europäischer Gesundheitssysteme. Studien zeigen, dass etwa 2 Drittel der ambulanten Eingriffe in Deutschland in freien Einrichtungen stattfinden, während nur ein Drittel von Krankenhäusern erbracht wird [49]. Als wesentliche Barrieren gelten nach wie vor unterschiedliche Vergütungslogiken und die strikte Trennung zwischen ambulantem und stationärem Sektor [48]. So sind stationäre Eingriffe im DRG-System oft finanziell attraktiver als eine ambulante Durchführung und begründeten so über die zurückliegenden Jahre diverse Fehlanreize [50].

Ein Blick auf internationale Modelle zeigt, dass eine konsequente Förderung ambulanter Eingriffe nicht nur Kostenvorteile bringt, sondern auch Patientenzufriedenheit steigern kann [51]. In Skandinavien etwa, v. a. in Dänemark und Norwegen, sind elektive Eingriffe fast vollständig als „same-day surgery“ organisiert [52]. Hier sorgt ein Vergütungssystem, das ambulante und stationäre Leistungen gleich bewertet, dafür, dass keine Fehlanreize entstehen. Die Ambulantisierungsraten sind entsprechend hoch, wenngleich eine sichere Indikationsstellung und eine strukturierte postoperative Betreuung entscheidend bleiben.

Auch im englischen National Health Service (NHS) wurde eine deutliche Verlagerung durch finanzielle Anreize erreicht. Mit den „best practice tariffs“ erhielten Kliniken höhere Vergütungen, wenn bestimmte Eingriffe ambulant durchgeführt wurden. Damit gelang es, die Zahl der stationären Behandlungen für standardisierbare Operationen deutlich zu senken. Grenzen des Modells zeigen sich allerdings in den bekannten Wartelistenproblemen und einer chronischen Ressourcenknappheit des NHS [53]. Die Niederlande und die Schweiz haben sich für Hybridmodelle entschieden, bei denen ambulante Eingriffe entweder in Krankenhäusern oder in spezialisierten Zentren erbracht werden [54, 55]. In der Schweiz gilt seit 2019 zudem eine Pflicht, bestimmte Eingriffe ambulant durchzuführen, wobei flankierende Tarifänderungen eingeführt wurden, um Kliniken nicht zu benachteiligen. Erste Auswertungen zeigen, dass so Kosten gesenkt und gleichzeitig die Versorgungsqualität stabil gehalten werden konnten [55].

Ein anderes Modell findet sich in Australien und Teilen Kanadas. Dort übernehmen große ambulante Op.-Zentren, oft in privater Trägerschaft, einen Großteil der elektiven Eingriffe. Diese Einrichtungen sind hoch spezialisiert, was Effizienzgewinne ermöglicht und Wartezeiten verkürzt. Allerdings zeigen sich dort auch Herausforderungen bei der flächendeckenden Versorgung und beim Zugang für sozioökonomisch benachteiligte Patientengruppen [56, 57].

Was lässt sich aus diesen Erfahrungen für Deutschland ableiten? Erstens bedarf es einer Anpassung der Vergütung. Solange Krankenhäuser mit stationären Eingriffen mehr Erlöse erzielen als mit ambulanten, bleibt eine echte Verlagerung unrealistisch. Modelle mit sektorengleicher Vergütung, wie sie in der Schweiz oder in Skandinavien erprobt wurden, könnten hier als Vorbild dienen. Zweitens muss die Qualitätssicherung für ambulante Operationen in Praxen und freien Op.-Zentren konsequent gewährleistet werden. Untersuchungen zeigen, dass bei standardisierten Abläufen und klaren Hygiene- und Sicherheitsvorgaben die Ergebnisse in freien Zentren ebenso gut oder sogar besser sein können als in Krankenhäusern. Drittens braucht es strukturelle Flexibilität. Interdisziplinäre medizinische Versorgungszentren könnten Ressourcen bündeln und so auch komplexere Eingriffe ambulant abbilden. Day-Surgery-Abteilungen innerhalb von Krankenhäusern bieten zusätzlich die Möglichkeit, stationäre Kapazitäten zu entlasten und gleichzeitig Sicherheit für Risikopatienten zu gewährleisten.

Regulatorisch sind klare Rahmenbedingungen notwendig. Ein verbindlicher Katalog ambulant durchzuführender Eingriffe existiert zwar, muss aber erweitert und gleichzeitig mit einer angemessenen Finanzierung verknüpft werden. Dabei darf die Patientensicherheit nicht aus dem Blick geraten. Nicht jeder Patient eignet sich für ambulantes Operieren, und eine gute Auswahl nach Alter, Komorbiditäten und sozialer Situation bleibt essenziell [58, 59]. Auch Nachsorgekonzepte wie eine 24-h-Erreichbarkeit im Komplikationsfall sind unverzichtbar [60].

Zusammenfassend zeigt der internationale Vergleich, dass Deutschland viel Potenzial hat, die Ambulantisierung voranzutreiben (Tab. 2). Skandinavische Länder demonstrieren, wie einheitliche Vergütungen Fehlanreize beseitigen können, England macht die Wirkung gezielter Tarifanreize sichtbar, die Schweiz belegt den Erfolg einer gesetzlichen Pflicht zur ambulanten Durchführung bestimmter Eingriffe, und Australien sowie Kanada zeigen, welche Effizienz spezialisierte Zentren erreichen können. Für Deutschland wird es entscheidend sein, finanzielle und regulatorische Barrieren abzubauen, die Strukturen flexibler zu gestalten und die Qualitätssicherung konsequent weiterzuentwickeln. Nur so kann die Balance zwischen operativer Tätigkeit in Klinik und Praxis neu austariert werden – im Sinne von Patientenorientierung, Wirtschaftlichkeit und Versorgungssicherheit.

**Tab. 2:** Ländervergleich der Ambulantisierung in der HNO-Heilkunde

Land/Modell	Merkmale/Strategien	Vorteile/Herausforderungen
Skandinavien (DK, NO, SE)	Viele Eingriffe als „same-day surgery“; ambulant und stationär gleich vergütet	Hohe Ambulantisierungsrate, geringere Kosten; Herausforderung: Qualität und Nachsorge
England (NHS)	„Best practice tariffs“ mit Anreizen für ambulante Durchführung	Verlagerung gelungen; Limitation: Wartelisten, Ressourcenknappheit
Niederlande/Schweiz/FR	DRG-ähnliche oder hybride Systeme; in CH Pflicht für bestimmte ambulante Eingriffe	Flexibilität und Zugang; Herausforderung: komplexe Abrechnung, Standards
Australien/Kanada	Große spezialisierte Op.-Zentren, teils privat; sektorübergreifende Tarife	Effizienz, kürzere Wartezeiten; Herausforderung: regionale Unterschiede, Übertragbarkeit

*DRG *Diagnosis Related Groups,* NHS* National Health Service

## „Green surgery“ – Nachhaltigkeit im HNO-OP

Das Thema Nachhaltigkeit gewinnt auch im chirurgischen Bereich zunehmend an Bedeutung. Der Gesundheitssektor verursacht in Deutschland rund 5 % der nationalen CO_2_-Emissionen [61], wobei Operationssäle zu den ressourcenintensivsten Bereichen zählen [62]. Energieverbrauch, Materialeinsatz und Abfallaufkommen liegen hier deutlich über den Durchschnittswerten anderer Krankenhausbereiche. Der Begriff „green surgery“ beschreibt Strategien, die ökologische Verantwortung in den chirurgischen Alltag integrieren, ohne die Patientensicherheit zu gefährden [63].

### Ökologischer Fußabdruck des Operierens

Ein einzelner chirurgischer Eingriff kann – je nach Dauer, Materialeinsatz und Anästhesieverfahren – mehrere Kilogramm CO_2_-Äquivalente verursachen [64]. Hauptquellen der Emissionen sind:

- Energieverbrauch (insbesondere durch Beleuchtung, Lüftungsanlagen, Klimatisierung und Sterilisation),
- Einwegprodukte und Verpackungsmaterialien,
- Narkosegase wie Desfluran und Lachgas, die ein erhebliches Treibhauspotenzial besitzen, sowie
- Abfallentsorgung und Transportwege in der Materiallogistik [64, 65].

In der HNO-Chirurgie fallen insbesondere bei ambulanten und mikroskopisch-endoskopischen Eingriffen große Mengen an Einweginstrumenten und Abdeckmaterial an. Der Anteil wiederverwendbarer Instrumente ist zwar gestiegen, wird aber oft durch regulatorische und hygienische Anforderungen begrenzt [66].

### Energie- und Ressourcenmanagement

Ein zentraler Ansatz der „green surgery“ ist die Energieoptimierung von Op.-Bereichen. Lüftungs- und Klimaanlagen sind für bis zu 60 % des Energieverbrauchs eines Op.-Saals verantwortlich [67]. Moderne, bedarfs-/belegungsabhängige Steuerungen können den Luftaustausch und die Klimatisierung außerhalb aktiver Op.-Zeiten deutlich reduzieren [67, 68]. Auch der Einsatz energieeffizienter LED-Beleuchtung und intelligenter Steuerungssysteme trägt zur Reduktion des Stromverbrauchs bei [68].

Ein weiterer Hebel ist die Optimierung der Sterilgutlogistik. Die Reduktion von Übersterilisationen, der gezielte Einsatz von Mehrwegtextilien und die Bündelung von Op.-Sets nach realem Verbrauch senken nicht nur Materialkosten, sondern auch CO_2_-Emissionen [69].

### Nachhaltige Material- und Abfallstrategien

In der HNO-Chirurgie entstehen große Mengen an Verpackungsmüll, insbesondere durch Einwegendoskope, Sauger und Abdecksysteme [70]. Nachhaltige Konzepte setzen auf:

- Mehrweg- und Recyclinglösungen, sofern technisch und hygienisch vertretbar,
- Materialien aus recycelbaren Kunststoffen sowie
- die Getrenntsammlung von Abfällen, um Recyclingquoten zu erhöhen [70, 71].

Pilotprojekte zeigen, dass durch strukturierte Abfalltrennung im OP bis zu 30 % des Restmülls in den Wertstoffkreislauf zurückgeführt werden kann [71].

### Anästhesie und Klimaschutz

Ein oft unterschätzter Faktor sind die Treibhauspotenziale von Inhalationsanästhetika. Gase wie Desfluran besitzen ein mehr als 2000-fach höheres Treibhauspotenzial als CO_2_ [72]. Durch den Wechsel auf total intravenöse Anästhesie (TIVA) oder den Einsatz von Sevofluran mit geringeren Emissionswerten lassen sich die CO_2_-Äquivalente eines Eingriffs erheblich reduzieren [73]. Anästhesieteams spielen damit eine Schlüsselrolle für klimabewusstes Operieren.

### Ambulantes Operieren als Nachhaltigkeitsfaktor

Die Verlagerung geeigneter Eingriffe in den ambulanten Bereich kann ebenfalls zur Emissionsminderung beitragen. Kürzere Aufenthalte bedeuten weniger Energie- und Wasserverbrauch, weniger Transport und geringeren Ressourcenbedarf für Unterkunft und Verpflegung [74]. Studien belegen, dass ambulante Eingriffe – bei gleicher Sicherheit – bis zu 70 % weniger CO_2_-Emissionen pro Fall verursachen als stationäre Operationen [75].

Gerade in der HNO-Chirurgie, wo viele Eingriffe ambulant durchführbar sind, eröffnet dies ökologische und ökonomische Synergien [74, 75].

### Zukunftsperspektiven: nachhaltige Op.-Innovation

„Green surgery“ erfordert ein Zusammenspiel technischer, organisatorischer und kultureller Veränderungen: Digitale Dokumentation und Prozesssteuerung können Materialverbrauch sichtbar machen und Optimierungspotenziale aufzeigen. Herstellerseitig müssen medizinische Geräte und Verbrauchsmaterialien stärker auf Lebenszyklusanalysen und Recyclingfähigkeit ausgerichtet werden. Ausbildung und Bewusstseinsbildung sind entscheidend, um Nachhaltigkeit als festen Bestandteil chirurgischer Qualität zu verankern.

Internationale Beispiele – etwa aus Großbritannien und Skandinavien – zeigen, dass klare Zielvorgaben („net zero surgery“) und verpflichtende Nachhaltigkeitsberichte die Entwicklung erheblich beschleunigen [76, 77].

Fazit: Nachhaltigkeit im OP ist keine Randerscheinung, sondern wird zunehmend zum Qualitätsmerkmal chirurgischer Versorgung.

Für die HNO-Chirurgie bedeutet „green surgery“, den ökologischen Fußabdruck zu kennen, gezielt zu reduzieren und die Balance zwischen technischer Innovation, Patientensicherheit und Umweltverantwortung zu wahren.

Ambulante und stationäre HNO-Eingriffe können durch bewussten Materialeinsatz, energieeffiziente Abläufe und klimafreundliche Anästhesie einen messbaren Beitrag zum Klimaschutz leisten – ohne Abstriche bei medizinischer Qualität.

## Zukunft der Aus- und Weiterbildung

Wie sieht die Zukunft in der Aus- und Weiterbildung in der operativen HNO-Heilkunde aus? Die operative Aus- und Weiterbildung in der HNO-Heilkunde steht in Deutschland vor einem strukturellen Shift: Mit Krankenhausreform (KHVVG), AOP-Ausbau und Hybrid-DRG verlagert sich ein wachsender Teil der „Bread-and-Butter-Eingriffe“ in den ambulanten Sektor, während komplexe und hochspezialisierte Leistungen stärker in Zentren gebündelt werden [78, 79]. Für die Weiterbildung bedeutet das nicht „Praxis oder Klinik“, sondern ein planvolles Sowohl-als-auch – mit klarer Profilbildung der Lernorte [80]. Seit 1. Januar 2025 gilt der an den OPS 2025 angepasste AOP-Katalog; zahlreiche HNO-Prozeduren sind explizit ambulant abzurechnen [78]. Parallel zeigen GKV-Daten einen markanten Mengenzuwachs ambulanter HNO-Operationen bereits 2023 (z. B. +44 % gegenüber 2022), was die Verschiebung der Eingriffsrealität für Ärzte/Ärztinnen in Weiterbildung (AiW) unterstreicht [78, 79]. Hybrid-DRG wurden 2024 eingeführt und sollten für die HNO-Heilkunde erweitert werden; sie schaffen sektorengleiche Vergütung für ausgewählte Eingriffe und erleichtern tagesstationäre/ambulante Durchführung – wiederum mit Relevanz für operative Lerngelegenheiten außerhalb des stationären Settings [79, 81].

Im stationären Bereich setzt die Reform gleichzeitig auf Qualitätssteuerung über Leistungsgruppen und verbindliche Strukturprüfungen. Für die HNO-Heilkunde heißt das: Maximal- und Schwerpunktversorger – häufig Universitätskliniken – erfüllen die hohen Struktur- und Prozessanforderungen und halten hochkomplexe Eingriffe (z. B. onkologische Kopf-Hals-Chirurgie, Schädelbasis- und Cochlea-Implantat-Versorgung) vor; periphere Häuser verlieren solche Leistungen tendenziell, wenn die Kriterien oder Zuweisungen fehlen. Für die Weiterbildung ist die Konsequenz klar: komplexe Kompetenzentwicklung bleibt klinikzentriert, während das operative Basisspektrum systematisch im ambulanten Umfeld erlernt und konsolidiert werden sollte. Aber wie soll die operative Ausbildung dann noch funktionieren? Junge Assistenzärzte beginnen meistens ihre Ausbildung in den Kliniken mit operativen Eingriffen wie der Adenotomie, der Tonsillektomie, Septumplastiken usw. Werden diese Eingriffe künftig noch in den Kliniken angeboten? Wäre es nicht viel sinnvoller, wenn die chirurgische Ausbildung dann teilweise in der Klinik und teilweise in ambulanten Op.-Zentren/MVZ-Strukturen usw. stattfindet? Hier scheint ein nicht unerhebliches Potential gerade für die chirurgische Ausbildung zu liegen.

Regulatorisch stützt die (Muster‑)Weiterbildungsordnung (MWBO, Fassung 03.07.2025) den Kompetenzansatz mit Logbuch-/eLogbuch-Dokumentation [80, 82]. Landesärztekammern präzisieren die Umsetzbarkeit im ambulanten Setting; inzwischen sind je nach Praxis und Bundesland auch große Teile der Weiterbildung in der Praxis möglich, eingebettet in die insgesamt 5‑jährige Facharztweiterbildung [83]. Digitale Dokumentation (eLogbuch) ist inzwischen Standard [82]. Die durch die Krankenhausreform veränderten Rahmenbedingungen könnten die sektorenübergreifenden Rotationen in der Weiterbildung begünstigen: Klinikjahre für Notfall‑, Intensiv‑, Tumor- und Revisionschirurgie; Praxis‑/Ambulanzmonate für Adenotomie/Tonsillotomie, Septum‑/Rhinologie-Basics, Myringotomie mit Paukenröhrchen, kleine Ohr- und Speicheldrüseneingriffe, perioperative Sprechstunde, Nachsorge und Komplikationsmanagement [80, 83].

Finanziell und organisatorisch wird die ambulante Weiterbildung zusätzlich erleichtert: Nach § 75a SGB V stehen bundesweit geförderte Weiterbildungsstellen im vertragsärztlichen Bereich zur Verfügung; die monatliche Förderung für AiW in Praxen liegt (je Vollzeit) aktuell bei 5800 €. Regionale Richtlinien spezifizieren Kontingente und Arztgruppen [84, 85]. Hierbei ist die HNO-Heilkunde ist in den Regionen vieler Kassenärztlicher Vereinigungen ausdrücklich förderfähig. Damit wird es realistischer, größere Teile des operativen Basiskatalogs in MVZ/Beleg-Op.-Zentren zu erlernen, ohne auf tarifnahe Vergütung zu verzichten; allerdings ist die Beantragung der Fördermittel mit einem hohen bürokratischen Aufwand verbunden, und die Erfahrung zeigt, dass mit den Fördermitteln nicht fest gerechnet werden kann.

Didaktisch verschiebt sich der Fokus in Richtung kompetenzbasierter Curricula mit standardisierten Rotationen, Op.-Richtzahlen und Simulation (Mikrochirurgie-Box-Trainer, Endoskopie-Skills, Lappenpräparation im Präparierkurs) und E‑Learning (z. B. HNO-eCampus der DGHNO-KHC, teilweise angeboten in der „HNO-App“) – eine notwendige Antwort auf kleinteiligere Op.-Zeiten im ambulanten Setting und auf die Zentralisierung komplexer Fälle. Fachgesellschaften und Kliniken bieten hierfür strukturierte Kursprogramme, die gezielt operative Teilkompetenzen adressieren (Rhinologie, Otologie, Lappenplastiken, Mikrovaskulartechnik) [80, 86].

Wo erhalten Assistenzärzte in Zukunft die bessere Ausbildung – in der Praxis oder in der Klinik? Kurz- bis mittelfristig wird der operative HNO-Nachwuchs beides brauchen: Kliniken (insbesondere universitäre und große Schwerpunktzentren) bleiben essenziell für komplexe Chirurgie, interdisziplinäre Tumorboards, Intensiv- und Notfallsituationen sowie für die individuelle wissenschaftliche Qualifikation. Praxen/MVZ und ambulante Op.-Zentren könnten zum primären Lernort für standardisierte Eingriffe mit hoher Frequenz und für das gesamte perioperative Management im ambulanten Setting werden. Durch die Qualitätssicherung im Rahmen der leistungsorientierten Patientenstruktur (Leistungsgruppenprüfungs-Richtlinie für OPS-Codes, LOPS) und strukturorientierten Patientenstruktur (Strukturprüfungs-Richtlinie für OPS-Codes, StrOPS) werden bundesweit einheitliche Mindeststandards für Struktur- und Leistungsqualität in den Kliniken sichergestellt. Die AOP- und Hybrid-DRG-Regelungen schaffen dagegen echte Lernkurven im ambulanten Alltag. Weiterbildungsbefugte in Klinik und Praxis müssen sich dann als verbindliche „Verbundweiterbildung“ strukturieren mit aufeinander abgestimmter Fall- und Kompetenzzuordnung, Op.-Katalog, im Idealfall kombiniert mit Supervisions- und Feedbackkultur sowie dokumentierten Rotationszielen – damit die Ärzt:innen in Weiterbildung (ÄiW) die ganze Breite der operativen HNO-Heilkunde trotz Sektorengrenzen abdecken. Voraussetzung hierfür sind aber verlässliche finanzielle Förderzusagen auch für die Weiterbilder in MVZ- und Praxisstrukturen, damit eine operative Ausbildung in einem Rotationsmodell mit den Kliniken durchgeführt und angeboten werden kann.

## Fazit für die Praxis

- Die operative Versorgung an HNO-Universitätskliniken, peripheren Häusern und Belegärztlichen Einrichtungen ist essenziell für eine qualitativ hochwertige, flächendeckende und innovative Patientenversorgung.
- Doch ökonomischer Druck, Fachkräftemangel und teils veraltete Strukturen stellen diesen Versorgungsauftrag zunehmend infrage.
- Die Krankenhausreform bietet grundsätzlich Chancen – insbesondere durch eine stärkere Strukturierung und differenzierte Leistungsplanung.
- Ob Universitätskliniken jedoch dauerhaft gestärkt und periphere Häuser sowie ambulant tätige Ärztinnen und Ärzte geschwächt daraus hervorgehen, hängt entscheidend davon ab, ob ihre besonderen Aufgaben im Bereich Hochleistungsmedizin, Forschung und Ausbildung realistisch abgebildet und finanziell getragen werden.
- Die operative HNO-Chirurgie nicht nur an Unikliniken braucht gezielte politische, strukturelle und finanzielle Unterstützung – nicht nur zur Reform, sondern für die Versorgung der Zukunft.
- Universitätskliniken und periphere Kliniken mit breitem Behandlungsspektrum sichern unter der Krankenhausreform die Maximalversorgung (einschließlich Cochleaimplantaten [CI]) und profitieren von Vorhaltevergütung sowie planungsrechtlicher Priorisierung; kleinere periphere Kliniken bleiben wichtige Anbieter grundständiger HNO-Leistungen, rücken aber – befördert durch AOP- (Ambulantes Operieren im Krankenhaus) und Hybrid-DRG-Regelungen (Diagnosis Related Groups) – stärker in den ambulanten bzw. kurzstationären Bereich.
- Landesplanerische Entscheidungen (NRW, Bayern) und die Prüfsystematik des Medizinischen Diensts (MD) sorgen dafür, dass HNO-Leistungen mit hohen Struktur- und Qualitätsanforderungen (z. B. Cochleaimplantat [CI], onkologische Kopf-Hals-Chirurgie) konzentriert vorgehalten werden, während routinefähige Eingriffe regional verteilt und vermehrt ambulant erbracht werden.
- Es bleibt die Frage, wie und wo die Ärzt:innen in Weiterbildung (ÄiW) ihre Ausbildung und Op.-Zahlen insbesondere für die kleineren Operationen sammeln sollen und ob hierfür mehr Kooperation in der Weiterbildung sinnvoll wäre.
- Für die Versorgung im ländlichen Raum bleibt die Kooperation zwischen Universitätskliniken (Zentrumsleistungen, Forschung/Lehre) und peripheren Kliniken/Medizinischen Versorgungszentren (MVZ; ambulante Op.-Kapazitäten, Nachsorge) zentral – mit der Perspektive, dass nicht zugewiesene periphere HNO-Stationen mittelfristig in ambulatorische Leistungsangebote überführt werden könnten.

## References

[1] Deutsches Ärzteblatt (2025) So weit sind die Bundesländer bei der Umsetzung der Krankenhausreform. https://www.aerzteblatt.de/news/so-weit-sind-die-bundeslander-bei-der-umsetzung-der-krankenhausreform-581b0e3f-acea-4085-a819-72e9486f2693

[2] Löhler J, Delank W, Drumm S et al (2022) Erwägungen zur Durchführung und Qualitätssicherung ambulanter Operationen im HNO-Bereich in Deutschland. Laryngorhinootologie 101:866–875 36257337 10.1055/a-1946-1458

[3] IGES Institut (2022) Gutachten oder auch Endbericht zum Projekt „Ambulantisierungspotenziual in Deutschen Akutkrankenhäusern“. IGES_AOP (Ambulantes Operieren im Krankenhaus) (Korrigierter Bericht vom 15.02.2024)

[4] Deitmer T, Dietz A, Delank KW et al (2021) Ambulantes Operieren in der HNO-Heilkunde in Deutschland. Laryngorhinootologie 101:464–483 33822330 10.1055/a-1418-9745

[5] Hoffmann TK, Deitmer T, Lippert BM, Datzmann T, Drumm S, Löhler J (2024) Stellungnahme der Wissenschaftlichen Fachgesellschaft und des Berufsverbandes zur Notfallversorgung in der Hals-Nasen-Ohren-Heilkunde. Laryngorhinootologie 103:578–585 38917833 10.1055/a-2338-0808

[6] Sennewald C Stand der Umsetzung der neuen Krankenhausplanung in Nordrhein-Westfalen. Dialogforum für Leitende Ärztinnen Ärzte am 28 Mai 2024. https://www.aekno.de/fileadmin/user_upload/aekno/downloads/2024/dialogforum-2024-01.pdf

[7] Anhörung Landesteil Nordrhein – u. a. Leistungsgruppe 24.2 Cochleaimplantate. Düsseldorf: MAGS NRW; 2024.

[8] BVHNO Thomas Hahn (2025) Brandbrief an Politik und Kassen: HNO-Kinderchirurgie steht vor dem Kollaps. https://www.hno-aerzte.de/presse/pressemitteilungen/details/brandbrief-an-politik-und-kassen-hno-kinderchirurgie-steht-vor-dem-kollaps/. Zugegriffen: 14. Okt. 2025

[9] Kassenärztliche Bundesvereinigung (KBV) (2025) Einheitlicher Bewertungsmaßstab (EBM). Berlin

[10] Deutsche Gesellschaft für Hals-Nasen-Ohren-Heilkunde, Kopf- und Hals-Chirurgie (DGHNO-KHC) (2022) Ambulantes Operieren in der HNO – Positionspapier

[11] Bundesärztekammer (BÄK) (2021) Belegärztliche Tätigkeit: Chancen und Herausforderungen. Dtsch Ärztebl 118(46):A-2236

[12] Bundesministerium für Gesundheit (BMG) (2022) Gesetz zur Weiterentwicklung der Gesundheitsversorgung (GVWG) und Ambulantisierung: Ambulant vor stationär. https://www.bundesgesundheitsministerium.de/service/gesetze-und-verordnungen/detail/gesundheitsversorgungsweiterentwicklungsgesetz-gvwg.html. Zugegriffen: 14. Okt. 2025

[13] Bundesärztekammer (2023) Kommentar zur Gebührenordnung für Ärzte (GOÄ)

[14] InEK GmbH (2024) Fallpauschalen-Katalog

[15] Siegert R Abrechnung medizinischer Leistungen: Eine Übersicht. Hno Informationen 0225(02) (Aus den Reihen der Mitglieder)

[16] Medizinischer Dienst Bund (2024) IGeL Monitor

[17] Deutscher Berufsverband der Hals-Nasen-Ohrenärzte e. V. (2023) Stellungnahme des Deutschen Berufsverbandes der Hals-Nasen-Ohrenärzte e. V. zum Referentenentwurf einer Verordnung zu einer speziellen sektorengleichen Vergütung (Hybrid-DRG-V)

[18] BMG Verordnung zu einer speziellen sektorengleichen Vergütung (Hybrid-DRG-V). https://www.bundesgesundheitsministerium.de/service/gesetze-und-verordnungen/detail/hybrid-drg-v.html. Zugegriffen: 14. Okt. 2025

[19] Deutsche Krankenhausgesellschaft e. V. (2023) Stellungnahme der Deutschen Krankenhausgesellschaft zum Referentenentwurf einer Verordnung zu einer speziellen sektorengleichen Vergütung (Hybrid-DRG-V)

[20] Liu Y, Wu X, Sang Y, Zhao C et al (2024) Evolution of surgical robot systems enhanced by artificial intelligence: a review. Adv Intell Syst 6(5):2300268

[21] Cheikh Youssef S, Haram K, Noël J et al (2023) Evolution of the digital operating room: the place of video technology in surgery. Langenbecks Arch Surg 408(1):95 36807211 10.1007/s00423-023-02830-7 PMC9939374

[22] Aweeda M, Adegboye F, Yang SF, Topf MC (2024) Enhancing surgical vision: augmented reality in otolaryngology—head and neck surgery. J Med Ext Real 1(1):124–136 39091667 10.1089/jmxr.2024.0010 PMC11290041

[23] Nobre ML, Sarmento ACA, de Bedaque HP et al Image guidance for endoscopic sinus surgery: systematic review and meta-analysis. Rev Assoc Médica Bras 69(10):e20230633 10.1590/1806-9282.20230633 PMC10578316 37851719

[24] Neves CA, Tran ED, Kessler IM, Blevins NH (2021) Fully automated preoperative segmentation of temporal bone structures from clinical CT scans. Sci Rep 11(1):116 33420386 10.1038/s41598-020-80619-0 PMC7794235

[25] Wang J, Wang J, Ma F et al (2021) Fully automated segmentation in temporal bone CT with neural network: a preliminary assessment study. BMC Med Imaging 21:166 34753454 10.1186/s12880-021-00698-x PMC8576911

[26] Gatto A, Tofanelli M, Costariol L et al (2023) Otological planning software—OTOPLAN: a narrative literature review. Audiol Res 13(5):791–801 37887851 10.3390/audiolres13050070 PMC10603892

[27] Jerosha S, Subramonian SG, Mohanakrishnan A et al Preoperative assessment using CT and MRI scans of the temporal bone to determine the degree of difficulty in cochlear implant surgery. Cureus 16(7):e65196 10.7759/cureus.65196 PMC11341111 39176341

[28] Puel U, Beysang A, Hossu G et al (2025) Comparison of CT-like MRI sequences for preoperative planning of cochlear implantation using super-high-resolution CT as a reference. Eur Radiol Exp 9(1):1 39747801 10.1186/s41747-024-00538-x PMC11695506

[29] Lee WJ, Kim YH, Hong SD et al (2022) Development of 3‑dimensional printed simulation surgical training models for endoscopic endonasal and transorbital surgery. Front Oncol 12:10.3389/fonc.2022.966051 PMC9389537 35992880

[30] Wu BZ, Hu LH, Cao SF et al (2025) Deep learning-based multimodal CT/MRI image fusion and segmentation strategies for surgical planning of oral and maxillofacial tumors: a pilot study. J Stomatol Oral Maxillofac Surg 126(Suppl):102324 40174752 10.1016/j.jormas.2025.102324

[31] O’Hara JT, Hurt CN, Ingarfield K et al (2024) Transoral laser or robotic surgery outcomes for oropharyngeal carcinoma: secondary analysis of the PATHOS randomized clinical trial. JAMA Otolaryngol Head Neck Surg 150:1002–1011 39388196 10.1001/jamaoto.2024.3371 PMC11581722

[32] Caversaccio M, Wimmer W, Anso J et al (2019) Robotic middle ear access for cochlear implantation: first in man. PLoS ONE 14(8):e220543 31374092 10.1371/journal.pone.0220543 PMC6677292

[33] Lang S, Mattheis S, Hasskamp P et al (2017) A european multicenter study evaluating the flex robotic system in transoral robotic surgery. Laryngoscope 127(2):391–395 27783427 10.1002/lary.26358

[34] Yamamoto N, Okano T, Yamazaki H et al (2019) Intraoperative evaluation of cochlear implant electrodes using mobile cone-beam computed tomography. Otol Neurotol 40(2):177–183 30624399 10.1097/MAO.0000000000002097

[35] Catanzaro S, Copelli C, Manfuso A et al (2017) Intraoperative navigation in complex head and neck resections: indications and limits. Int J Comput Assist Radiol Surg 12(5):881–887 27659282 10.1007/s11548-016-1486-0

[36] Bartholomew RA, Zhou H, Boreel M et al (2024) Surgical navigation in the anterior skull base using 3‑dimensional endoscopy and surface reconstruction. JAMA Otolaryngol Neck Surg 150(4):318–326 10.1001/jamaoto.2024.0013 PMC11009826 38451508

[37] Lee YJ, Krishnan G, Nishio N et al (2021) Intraoperative fluorescence-guided surgery in head and neck squamous cell carcinoma. Laryngoscope 131(3):529–534 33593036 10.1002/lary.28822

[38] Badhey AK, Schwarz JS, Laitman BM et al (2023) Intraoperative use of wide-field optical coherence tomography to evaluate tissue microstructure in the oral cavity and oropharynx. JAMA Otolaryngol Neck Surg 149(1):71–78 10.1001/jamaoto.2022.3763 PMC9856682 36454583

[39] Mokhtar J, Almarzooqi S, Alhammadi F, Mendonca DA (2025) Pilot study: roboticscope (robotic microscope)—assisted primary cleft palate surgery. Plast Reconstr Surg Glob Open 13(5):e6744 40330164 10.1097/GOX.0000000000006744 PMC12055182

[40] Ajmera A, Campbell RG, Lee JWY, McGinness SJ, Mukherjee P (2025) Ergonomics of 3D-Exoscope versus the operating microscope in otologic surgery. ANZ J Surg 95(9):1862–1868 40757587 10.1111/ans.70278 PMC12484397

[41] Akbari L, Aarabi A, Bahrami M (2025) Challenges of Intraoperative documentation and its role in patient safety: an integrative review. Iran J Nurs Midwifery Res 30(2):141 40275930 10.4103/ijnmr.ijnmr_413_23 PMC12017641

[42] Watson JB, Quintero-Peña C, Moise AC et al From data to decision: a comprehensive review of real-time analytics and smart technologies in the surgical suite. Methodist Debakey Cardiovasc J 21(5):5–15 10.14797/mdcvj.1658 PMC12513365 41078851

[43] Georgenthum H, Cosentino C, Marozzo F, Liò P (2025) Enhancing surgical documentation through multimodal visual-temporal transformers and generative AI arxiv. http://arxiv.org/abs/2504.19918

[44] Arora A, Lau LYM, Awad Z, Darzi A, Singh A, Tolley N (2014) Virtual reality simulation training in otolaryngology. Int J Surg 12(2):87–94 24316019 10.1016/j.ijsu.2013.11.007

[45] Shahrezaei A, Sohani M, Taherkhani S, Zarghami SY (2024) The impact of surgical simulation and training technologies on general surgery education. BMC Med Educ 24(1):1297 39538209 10.1186/s12909-024-06299-w PMC11558898

[46] Kiyasseh D, Laca J, Haque TF et al (2023) A multi-institutional study using artificial intelligence to provide reliable and fair feedback to surgeons. Commun Med 3(1):42 36997578 10.1038/s43856-023-00263-3 PMC10063640

[47] OECD (2023) Ambulatory surgery: Health at a Glance 2023. https://www.oecd.org/en/publications/health-at-a-glance-2023_7a7afb35-en/full-report/ambulatory-surgery_f0b2228d.html. Zugegriffen: 15. Okt. 2025

[48] Deutscher Bundestag (2023) Vergleich von ambulanter und stationärer Versorgung in ausgewählten Ländern. Report No.: WD 9‑3000-017/23

[49] Brökelmann J (2012) Comparison of hospital- and office-based ambulatory surgery in Germany: surgery in small free standing units offers many advantages. Ambul Surg. 10.1016/j.ambsur.2003.09.006

[50] L M, Z O. International Comparison of Specialist Care Organization Innovations in Five Countries Germany. Interdisciplinary Ambulatory Healthcare Centers in Berlin Area (MVZs). Rapp Irdes. https://www.irdes.fr/english/2021/report-583-international-comparison-specialist-care-organization-germany-interdisciplinary-ambulatory-healthcare-centers.html (Erstellt: 15. Okt. 2021). Zugegriffen: 14. Okt. 2025 (583)

[51] OECD, European Union (2022) Health at a Glance: Europe 2022: State of Health in the EU Cycle. https://www.oecd.org/en/publications/health-at-a-glance-europe-2022_507433b0-en.html. Zugegriffen: 15. Okt. 2025

[52] Milstein R, Schreyögg J Activity-based funding based on diagnosis-related groups. The end of an era? A review of payment reforms in the inpatient sector in ten high-income countries. 10.1016/j.healthpol.2023.104990 38244342

[53] HS England, NHS Improvement (2021) Guidance on Best Practice Tariffs (National Tariff Payment System 2021/22). NHS England, London (https://www.england.nhs.uk/wp-content/uploads/2020/11/21-22NT_Annex-C-Best-practice-tariffs.pdf england.nhs.uk)

[54] Tulp ADM, Kruse FM, Stadhouders NW, Jeurissen PPT (2020) Independent treatment centres are not a guarantee for high quality and low healthcare prices in the Netherlands—A study of 5 elective surgeries. Int J Health Policy Manag 9(9):380–389 32610739 10.15171/ijhpm.2019.144 PMC7557426

[55] OBS The strategy to shift surgical interventions to outpatient settings has achieved its goals. https://eurohealthobservatory.who.int/monitors/health-systems-monitor/analyses (Erstellt: 15. Okt. 2025)

[56] Pace D, Hounsell C, Boone D (2023) Ambulatory surgery centres: a potential solution to a chronic problem. Can J Surg 66(2):E111–E113 36882204 10.1503/cjs.008022 PMC9998099

[57] DHA Profile (2021) Day hospitals Australia and the day hospital industry. https://www.dayhospitalsaustralia.net.au/wp-content/uploads/2021/11/DHA-Profile-2021.pdf?utm_source=chatgpt.com

[58] Rajan N, Rosero EB, Joshi GP (2021) Patient selection for adult ambulatory surgery: a narrative review. Anesth Analg 133(6):1415–1430 34784328 10.1213/ANE.0000000000005605

[59] Somani S, Capua JD, Kim JS et al (2017) ASA classification as a risk stratification tool in adult spinal deformity surgery: a study of 5805 patients. Glob Spine J 7(8):719–726 10.1177/2192568217700106 PMC5721995 29238634

[60] Daniels SA, Kelly A, Bachand D et al (2016) Call to care: the impact of 24-hour postdischarge telephone follow-up in the treatment of surgical day care patients. Am J Surg 211(5):963–967 26997305 10.1016/j.amjsurg.2016.01.015

[61] Fehrer V, Poß-Doering R, Weis A et al (2023) Climate change mitigation: Qualitative analysis of environmental impact-reducing strategies in German primary care. Eur J Gen Pract 29(1):2232946 37578422 10.1080/13814788.2023.2232946 PMC10431721

[62] Rizan C, Steinbach I, Nicholson R et al (2020) The carbon footprint of surgical operations: a systematic review. Ann Surg 272(6):986–995 32516230 10.1097/SLA.0000000000003951

[63] Dal Mas F, Cobianchi L, Piccolo D et al (2024) Are we ready for “green surgery” to promote environmental sustainability in the operating room? Results from the WSES STAR investigation. World J Emerg Surg 19(1):5 38267949 10.1186/s13017-024-00533-y PMC10809586

[64] Robinson P, Surendran K, Lim S, Robinson M (2023) The carbon footprint of surgical operations: a systematic review update. Ann R Coll Surg Engl 105(8):692–708 37906978 10.1308/rcsann.2023.0057 PMC10626532

[65] Samad K, Yousuf MS, Ullah H et al (2024) Anesthesia and its environmental impact: approaches to minimize exposure to anesthetic gases and reduce waste. Med Gas Res 15(1):101–109 39436173 10.4103/mgr.MEDGASRES-D-23-00059 PMC11515078

[66] Meiklejohn DA, Khan ZH, Nuñez KM et al (2024) Environmental impact of adult tonsillectomy: life cycle assessment and cost comparison of techniques. Laryngoscope 134(2):622–628 37421241 10.1002/lary.30866

[67] Mohamed A, Fuad U, Elsayed A, Elasad A Green surgery and sustainability in orthopaedic operating rooms: a comprehensive review of environmental initiatives. Cureus 17(8):e91369 10.7759/cureus.91369 PMC12488290 41041111

[68] Rovira-Simon J, Sales-i-Coll M, Pozo-Rosich P et al (2023) The green surgical block 4.0: automation of the operating theatre’s climate conditions through a real-time patient-flow solution. Future Healthc J 10(1):46–49 37786502 10.7861/fhj.2022-0142 PMC10538689

[69] Vozzola E, Overcash M, Griffing E An environmental analysis of reusable and disposable surgical gowns. Aorn J 111(3):315–325 10.1002/aorn.12885 32128776

[70] Ryan MT, Malmrose J, Riley CA, Tolisano AM (2023) Operating room waste generated across otolaryngology cases. Mil Med 188(7–8):usab548 34966924 10.1093/milmed/usab548

[71] Plezia D, Sabol VK, Nelson C, Simmons VC (2024) Improving waste segregation in the operating room to decrease overhead cost. Qual Manag Health Care 33(1):44–51 37296512 10.1097/QMH.0000000000000416

[72] Park EJ, Bae J, Kim J et al (2024) Reducing the carbon footprint of operating rooms through education on the effects of inhalation anesthetics on global warming: A retrospective study. Medicine 103(9):e37256 38428851 10.1097/MD.0000000000037256 PMC10906648

[73] Bernat M, Boyer A, Roche M et al (2024) Reducing the carbon footprint of general anaesthesia: a comparison of total intravenous anaesthesia vs. a mixed anaesthetic strategy in 47,157 adult patients. Anaesthesia 79(3):309–317 38205529 10.1111/anae.16221

[74] Tee NCH, Yeo JA, Choolani M, Poh KK, Ang TL (2024) Healthcare in the era of climate change and the need for environmental sustainability. Singapore Med J 65(4):204–210 38650058 10.4103/singaporemedj.SMJ-2024-035 PMC11132617

[75] Phull M, Begum H, John JB et al (2023) Potential carbon savings with day-case compared to inpatient transurethral resection of bladder tumour surgery in england: a retrospective observational study using administrative data. Eur Urol Open Sci 52:44–50 37284039 10.1016/j.euros.2023.03.007 PMC10240513

[76] Bhutta M, Rizan C (2024) The Green Surgery report: a guide to reducing the environmental impact of surgical care, but will it be implemented? Ann R Coll Surg Engl 106(6):475–477 38683381 10.1308/rcsann.2024.0005 PMC11214859

[77] Norwegian Directorate of Health Roadmap for sustainable, low-emission and climate-adapted health and care services. https://www.helsedirektoratet.no/rapporter/veikart-mot-en-baerekraftig-lavutslipps-og-klimatilpasset-helse-og-omsorgstjeneste

[78] Pioch C, Busse R, Mansky T, Nimptsch U (2025) The potential for providing treatment on an outpatient rather than inpatient basis: a nationwide analysis of hospital discharge data in Germany for the year 2022. Dtsch Arzteblatt Int 122(6):151–155 10.3238/arztebl.m2025.0012 PMC12456061 39908423

[79] Rathmayer M, Belle S, Heinlein W et al (2024) Impact analysis of a new, cross-sector service provision of gastroenterologic endoscopic services in accordance with 115f SGB V (hybrid-DRG): allocation matrix and cost analysis. Z Gastroenterol 62(5):705–722 38621703 10.1055/a-2292-9766

[80] Guntinas-Lichius O, Lang S, Stöver T et al (2023) Empfehlungen der Deutschen Gesellschaft für HNO-Heilkunde, Kopf- und Hals-Chirurgie (DGHNO-KHC) für die Erteilung der fachspezifischen Weiterbildungsbefugnis. Laryngorhinootologie 102:412–415 37267964 10.1055/a-2049-2576

[81] Kisch T, Müller-Rath R, Gregor S et al (2024) Hybrid diagnosis-related groups-The challenge. Chir Heidelb Ger 95(12):1007–1011 10.1007/s00104-024-02196-6 39505781

[82] Berberat PO, Harendza S, Kadmon M, Gesellschaft für Medizinische Ausbildung, GMA-Ausschuss für Weiterbildung (2013) Entrustable professional activities—visualization of competencies in postgraduate training. Position paper of the Committee on Postgraduate Medical Training of the German Society for Medical Education (GMA). GMS Z Med Ausbild 30(4):Doc47 24282450 10.3205/zma000890 PMC3839075

[83] Linke J, Eichhorn T, Kemper M, Zahnert T, Neudert M (2021) Die Weiterbildungssituation in der HNO-Heilkunde in Deutschland. HNO 69(7):534–543 34110437 10.1007/s00106-021-01068-3 PMC8233265

[84] Sierocinski E, Mathias L, Freyer Martins Pereira J (2022) Chenot JF. Postgraduate medical training in Germany: A narrative review. GMS J Med Educ 39(5):Doc49 36540556 10.3205/zma001570 PMC9733474

[85] Becker C, Stengel S, Roos M, Altiner A, Schwill S (2024) Starting postgraduate medical training in general practice with a rotation in general practice—a qualitative study on experiences and effects. GMS J Med Educ 41(5):Doc53 39711858 10.3205/zma001708 PMC11656185

[86] Bondzi-Simpson A, Lindo CJ, Hoy M, Lui JT (2022) The Otolaryngology boot camp: a scoping review evaluating commonalities and appraisal for curriculum design and delivery. J Otolaryngol Head Neck Surg 51:23 35659365 10.1186/s40463-022-00583-9 PMC9167522

